# Predictability of antigen binding based on short motifs in the antibody CDRH3

**DOI:** 10.1093/bib/bbae537

**Published:** 2024-10-22

**Authors:** Lonneke Scheffer, Eric Emanuel Reber, Brij Bhushan Mehta, Milena Pavlović, Maria Chernigovskaya, Eve Richardson, Rahmad Akbar, Fridtjof Lund-Johansen, Victor Greiff, Ingrid Hobæk Haff, Geir Kjetil Sandve

**Affiliations:** Department of Informatics, University of Oslo, Gaustadalléen 23B, 0373 Oslo, Norway; Department of Informatics, University of Oslo, Gaustadalléen 23B, 0373 Oslo, Norway; Department of Immunology, University of Oslo, Sognsvannsveien 20, Rikshospitalet, 0372 Oslo, Norway; Department of Informatics, University of Oslo, Gaustadalléen 23B, 0373 Oslo, Norway; Department of Immunology, University of Oslo, Sognsvannsveien 20, Rikshospitalet, 0372 Oslo, Norway; La Jolla Institute for Immunology, 9420 Athena Cir, La Jolla, CA, United States; Department of Immunology, University of Oslo, Sognsvannsveien 20, Rikshospitalet, 0372 Oslo, Norway; Department of Immunology, University of Oslo, Sognsvannsveien 20, Rikshospitalet, 0372 Oslo, Norway; Department of Immunology, University of Oslo, Sognsvannsveien 20, Rikshospitalet, 0372 Oslo, Norway; Department of Mathematics, University of Oslo, Niels Henrik Abels hus, Moltke Moes vei 35, 0851 Oslo, Norway; Department of Informatics, University of Oslo, Gaustadalléen 23B, 0373 Oslo, Norway

**Keywords:** adaptive immunology, antigen binding, computational immunology, motif discovery, machine learning

## Abstract

Adaptive immune receptors, such as antibodies and T-cell receptors, recognize foreign threats with exquisite specificity. A major challenge in adaptive immunology is discovering the rules governing immune receptor–antigen binding in order to predict the antigen binding status of previously unseen immune receptors. Many studies assume that the antigen binding status of an immune receptor may be determined by the *presence* of a short motif in the complementarity determining region 3 (CDR3), disregarding other amino acids. To test this assumption, we present a method to discover short motifs which show high precision in predicting antigen binding and generalize well to unseen simulated and experimental data. Our analysis of a mutagenesis-based antibody dataset reveals 11 336 position-specific, mostly gapped motifs of 3–5 amino acids that retain high precision on independently generated experimental data. Using a subset of only 178 motifs, a simple classifier was made that on the independently generated dataset outperformed a deep learning model proposed specifically for such datasets. In conclusion, our findings support the notion that for some antibodies, antigen binding may be largely determined by a short CDR3 motif. As more experimental data emerge, our methodology could serve as a foundation for in-depth investigations into antigen binding signals.

## Introduction

Adaptive immune receptors (AIRs) aid our body in defending itself against foreign threats by providing targeted recognition and neutralization of antigens. The most important site for antigen recognition is the highly variable complementarity determining region 3 (CDR3) of the antibody heavy chain or T-cell receptor (TCR) beta chain [1–3]. Therefore, many studies attempt to predict AIR-antigen binding exclusively based on the heavy/beta chain CDR3 sequences [4–8]. Yet, the underlying rules determining whether an AIR can bind an antigen of interest remain unknown [9–11].

The term *immune signal* has been introduced to describe the set of features associated with the antigen binding status of AIRs, or the disease status of an AIR repertoire (AIRR) [11, 12]. These features may reveal themselves in various dimensions of AIR(R) data, e.g.: amino acid motifs in the variable region of the AIRR sequence [13–15], biophysicochemical properties thereof [16, 17], 3D structural features [3, 18], or the clonal frequency distributions of AIRRs [19]. Machine learning (ML) algorithms can be used to learn immune signals in order to construct classifiers for antigen binding or disease prediction [4, 7, 8, 11, 20]. However, our lack of understanding of the shape and complexity of immune signals poses a major challenge in AIR(R)-ML [9–11]. The benefits of an improved understanding of the dimensions and parameters of immune signals are twofold. Firstly, it can guide the design of encodings and ML architectures that explicitly take into account the feature dimensions in which the immune signals are most pronounced, which may improve performance as well as interpretability of the ML models [21]. Secondly, it will aid us in making more informed AIR(R) data simulations, resulting in more thorough benchmarking of ML models, including testing their ability to recover ground truth simulated immune signals [11, 22].

Several studies have been performed under the assumption that AIR-antigen binding is determined by a short CDR3 motif, although this hypothesis has not been investigated directly. Our main research objective is to investigate to what degree antigen binding can be predicted based on the *presence* of short, possibly gapped, positional motifs in the antibody heavy chain CDR3, while varying the remaining positions of this CDR3 but keeping other variable regions fixed. Previous work addressing antigen binding associated CDR3 motifs primarily concerned TCR datasets. For example, short motifs have been used as the basis for clustering TCRs into antigen specificity clusters [13, 15]. Alternatively, some methods first cluster CDR3 sequences based on global similarity, and afterwards extract motifs enriched in clusters of sequences [23–25]. Such motifs can however always be found in a set of sequences with overall high similarity when compared to a more diverse reference set, and this does not confirm the sufficiency of these motifs for antigen binding. The assumption that short motifs determine antigen binding has also been extended to AIRR studies, where the disease state of TCR repertoires has been classified based on the presence of disease-associated k-mers [14, 16, 17, 26, 27], and AIRR disease states are commonly simulated through k-mer implanting [12, 28–31]. The use of 1D convolutional neural networks (CNNs) for the classification of individual AIRs or AIRRs [8, 32–36] also implies the assumption that (one or many) short, local patterns are of importance for determining antigen binding.

Furthermore, previous studies primarily considered local short motifs in the TCR CDR3 with either no [13, 15, 16] or few [17] gaps, as opposed to dispersed motifs, with the intent to capture contact residues (the paratope). More recently, antibody paratopes have also been found to be primarily contiguous or contain few gaps [3]. It should however be noted that the immune signal, if present in the form of short CDR3 motifs, does not necessarily correspond to the paratope, as mutations in non-contact residues may cause structural changes that substantially alter the binding affinity of the antibody [37–39]. In this case, the non-paratope residues are a crucial part of the immune signal. We therefore decided to consider not only contiguous but also highly dispersed motifs.

When investigating the factors affecting antibody–antigen binding, the choice of dataset is of crucial importance. For instance, it has previously been argued that global CDR3 sequence similarity can be used as a predictor of antigen binding status [5, 13, 25, 40]. However, the data distribution of blood-derived sequences is inherently skewed by biological processes, including V(D)J recombination, clonal expansion and somatic hypermutations. This may lead to the discovery of clusters of highly similar binders, whereas non-binders of similar sequence (that would fall in the same clusters) may simply not be observed due to lower V(D)J recombination probabilities or lack of selective signals. Such datasets thus primarily allow the discovery of rules reflecting sequence characteristics of immune responses [41], rather than rules specifically reflecting AIR-antigen binding. In contrast to datasets derived from natural AIR repertoires, mutagenesis allows to construct large sets of binder and non-binder CDR3 sequences that are both highly diverse and contain many examples of binders and non-binders differing in only one or few amino acid positions [32].

In this work, we investigate the presence of antigen binding-associated short motifs in a previously published HER2 binding combinatorial mutagenesis dataset [32]. We find such motifs, and show that they even generalize well to a second, independently generated mutagenesis dataset for the same target antigen [42]. Furthermore, combining these short motifs into a simple classifier allows to separate antigen binders from non-binders with a performance comparable to a deep learning model specifically developed for classifying AIR-antigen binding based on such mutagenesis datasets. We apply our method to antibody CDR3 datasets, and even though antigen binding rules may differ between TCRs and antibodies, this methodology for investigating binding rules may be applied in a similar way to TCRs.

## Material and methods

### A method to recover short CDR3 motifs associated with antigen binding

We are interested in the degree to which the presence of a CDR3 motif, composed of a combination of up to five position-specific amino acids with possible gaps, is sufficient for predicting that an AIR will bind a given antigen. We operationalize this as motifs that have high out-of-sample precision in separating antigen binders from non-binders. A high value of precision, also known as positive predictive value, means that when a motif is present in the CDR3, the probability of this AIR belonging to the binder class is high. However, the precision of high-ranked motifs on a given dataset provides a biased, over-optimistic estimate of their precision in the underlying AIR distribution of interest. When selecting motifs using a minimal precision threshold on a training dataset, the resulting motifs are overfitted to this training dataset and may not generalize well to new, unseen data. Under the standard assumption that the data are an independent and identically distributed (i.i.d.) sample from the underlying distribution, an unbiased precision estimate can be calculated on a separate dataset not considered during training (validation/test set).

**Figure 1:**
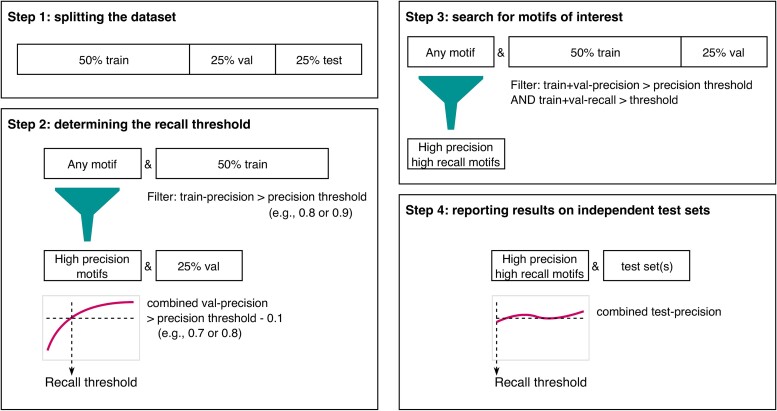
Workflow for finding motifs of interest. Step 1: The dataset is split into training, validation and test sets. Step 2: Using motifs with a training-precision greater than a chosen threshold, the appropriate recall threshold is selected at the point where the smoothed combined validation-precision reaches precision threshold – 0.1. This step is shown in more detail in Supplementary Fig. 1. Step 3: A new set of motifs is learned using data from the combined training and validation sets, by applying the chosen precision threshold and learned recall threshold. Step 4: Independent test data is used to report the performance of each motif in the final set of learned motifs.

The difference between the expected precision on the training dataset and unseen (validation/test) data, also known as generalization error, is strongly dependent on the number of antigen binding sequences containing the motif (true positives [TP]). This is because TP follows a binomial distribution *B(n, p)*, where *n* is the number of observed sequences containing the motif (true and false positives, TP + FP), and *p* is the true fraction of TP among the total set of theoretically possible sequences containing the motif. The observed motif precision in a given dataset is therefore an estimator of *p*, which has a large variance for small values of *n*. This high variance in precision for rare motifs results in a stronger tendency to overfit to the training data, and the high precision of such rare motifs is thus less likely to generalize well to unseen data. In order to discard all rare motifs for which generalizability is likely to be insufficient, we furthermore apply a learnable recall threshold to the motifs. We propose a workflow for finding antigen binding motifs with high precision and sufficient generalizability, and unbiased assessment of these motifs on a hold-out test set (Fig. 1).

We first divide our data into training, validation and test sets (Fig. 1 step 1). Next, the optimal recall thresholds for a given precision threshold are learned (Fig. 1 step 2). The total space of potential motifs is extremely large, and contains many amino acid combinations that do not occur in any sequence in the dataset. Thus, we employ an efficient algorithm to discover *all* candidate motifs of up to five amino acids long with at least two TPs in the training set. Our implementation is based on an itemset mining strategy analogous to the ‘candidate generation’ step of the a priori algorithm [43]. Briefly, each positional amino acid is considered an item, and its support is represented by its TP count. First, we consider all 20 amino acids in each position, and remove those with a TP count below 2. The remaining ‘legal’ positional amino acids will suffice as the building blocks of the motifs, and additionally represent the set of length-1 motifs. Next, each motif is recursively extended with any other ‘legal’ positional amino acid, so long as the TP count of the motif is at least 2. Motifs are extended on the right side only (with possible gaps) to reduce the search space and avoid duplicates. For each individual candidate motif, a precision score is computed on the training set, and a filter is applied to discard all motifs with a precision score below a given threshold (e.g., 0.8 or 0.9).

This initial set of high-training-precision motifs are used to calibrate a recall threshold (see Supplementary Fig. 1 for a more detailed graphical representation). The recall is the fraction of TPs (motif containing binders) among all positives (binders). We thus need to determine the minimum number of training set TPs required to discard rare motifs, which tend to have low validation-precision. We therefore consider at this step a *combined* precision on the validation set for each group of motifs with the same number of TPs on the training set. For a set of *n* motifs, the combined precision is defined as follows:


$$ {precision}_{combined}=\frac{\sum_{i=1}^n{TP}_i}{\sum_{i=1}^n{TP}_i+{\sum}_{i=1}^n{FP}_i}. $$

Note that when calculating the combined validation-precision for each training-TP group, this is still based on the TP and FP from the validation set. A crucial difference between the combined precision and average precision is that for combined precision, the FP predictions for motifs with 0 TP predictions (where precision is undefined) are still taken into account.

The combined validation precision curve is used to determine the number of TP, which is used as a basis to compute the recall threshold. Random variations in the data can cause this curve to be irregular, and smoothing of the curve is required to learn a stable estimate. This smoothing is done based on kernel smoothing using a log-normal kernel. The smoothing bandwidth is dynamically adapted based on the number of data points in a given window, resulting in stronger smoothing of parts of the curve where few data points are available. The recall threshold is calibrated to the point where the smoothed combined precision reaches the training precision threshold – 0.1. This subtraction of 0.1 reflects the fact that the training set precision is necessarily expected to be higher than the target precision for the underlying distribution, due to overfitting to the training set. Since the size of a motif has a large effect on the number of TPs (larger motifs are more unique than smaller motifs), recall thresholds are learned for each motif size independently.

The final set of motifs is extracted from the union of the training and validation sets by applying the same candidate motif generation algorithm, and filtering these motifs by the precision threshold and learned recall thresholds (Fig. 1 step 3). And finally, we use the test set to compute an unbiased estimate of the precision for each of the motifs (Fig. 1 step 4). The motif discovery method proposed here has been integrated into immuneML [20], a platform for comprehensive and reproducible ML-based analysis of AIR(R) data.

### Datasets

#### Experimental mutagenesis datasets

Two previously published combinatorial mutagenesis datasets of HER2 binder and non-binder CDR3 antibody heavy chain sequences were used in this work: the dataset by Mason et al. [32] as well as an independently generated experimental replicate, hereafter referred to as the Mehta dataset [42]. For the Mehta dataset, only samples 1 and 3 (strong and weak binders) were used. The antibodies in both these datasets use the same light chain, heavy chain V and J genes, and heavy chain CDR3 length, meaning that the only variability across the different antibodies is in 10 consecutive amino acid positions of the CDR3.

A custom Python script was created to preprocess the experimental data, which is available at https://github.com/LonnekeScheffer/short_motif. During preprocessing, duplicate sequence entries are aggregated. Overlapping sequences between binder and non-binder classes are sorted into the majority class if the count is more than twice as high as in the other class, and removed otherwise. See the supplementary materials for the dataset sizes after preprocessing (Supplementary Table 1), number of overlapping sequences (Supplementary Table 2), the positional frequency distributions of the Mason dataset (Supplementary Fig. 2) and Mehta dataset (Supplementary Fig. 3), as well as a comparison of amino acid usage between the two datasets (Supplementary Fig. 4).

#### Simulated datasets

To ensure our method works as expected, we applied it to two simulated datasets. These datasets represent extreme opposite cases, where in the first simulated dataset, antigen binding can be perfectly predicted by the presence of implanted ground truth motifs, whereas in the second dataset, no truly antigen-binding associated motifs are present. The Python script used to generate the synthetic datasets is available at https://github.com/LonnekeScheffer/short_motif.

Each simulated dataset consisted of 10.000 binder and 25.000 non-binder sequences of length 10, approximately matching the size of the preprocessed Mason dataset (Supplementary Table 1). The positional amino acid distribution of the synthetic datasets was initially matched to the distribution of the complete Mason dataset (binders and non-binders combined). Ten different ground truth motifs were selected for implanting in the first simulated dataset. Each motif is a combination of three position-specific amino acids with possible gaps in between. The positions in the sequence were randomly chosen, and the amino acids for these given positions were chosen according to their relative frequencies in the Mason dataset (Supplementary Fig. 5). This resulted in preferential use of ‘common’ amino acids, rather than disrupting the frequency distribution by inserting rare positional amino acids at high frequencies. In order to simulate both ‘rare’ and ‘common’ motifs, the motifs were inserted at varying frequencies ranging from 10 to 5005 with approximate 2-based logarithmic increments (Supplementary Table 3).

### ML classification of CDR3 sequences

We hypothesize that different motifs may be attributed to the antigen binding status of different CDR3s, and that it could therefore be possible to find a collection of complementary motifs which together can be used to classify the Mason test set (25%) and independent Mehta test set. We use the AIRR-ML platform immuneML [20] for the training and performance assessment of classifiers. For this study, several new classification algorithms were integrated into immuneML (Supplementary Table 4): motif-based classifiers, Hamming distance-based classifiers as well as the original CNN by Mason et al. [32].

A simple motif-based classifier maintains a set of motifs and predicts a sequence to be a binder if it contains any of the motifs in the set. We apply two versions of this classifier: *all motifs* uses the entire set of motifs learned on the Mason training + validation set, whereas *selected motifs* uses greedy forward selection to find a subset of motifs that maximizes balanced accuracy on the training set, until a drop in balanced accuracy on the validation set is observed for five consecutive motifs (early stopping with patience = 5). We furthermore consider a scikit-learn [44] logistic regression model which uses the full set of learned motifs as binary features. To select the optimal hyperparameters, 3-fold cross-validation is performed on the training set with balanced accuracy as optimization metric (Supplementary Table 5). We independently consider each of these three motif-based classifiers for motifs learned using precision thresholds 0.8 and 0.9.

The custom implemented Hamming distance-based classifier was included as a simple baseline model [45, 46]. This classifier stores all binders from the training + validation set, and predicts any sequence to be a binder if it is within a given Hamming distance (1, 2, or 3) from any stored binder sequence. For speed and memory efficiency, the sequence comparison is performed using CompAIRR [47], a tool for ultra-fast comparison of sets of CDR3 sequences with possible Hamming distance.

Mason et al. [32] focused primarily on the development of a CNN due to its high performance compared to other model types. The final CNN architecture comprises a convolutional layer followed by dropout, max pooling and finally a dense layer. The CNN algorithm was integrated into immuneML and trained on the training + validation set using the optimized hyperparameters from the original study (Supplementary Table 6).

## Results

### Recovering short antigen-binding associated motifs on simulated data

**Figure 2:**
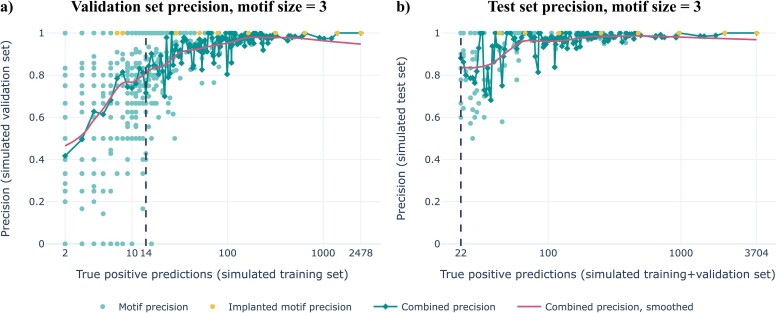
Our method can recover ground truth motifs on simulated data. (a) the validation set precision at different training-TP rates for motifs filtered with precision threshold 0.9 on the training set. The training-TP threshold is determined to be 14, as this is the point where the smoothed validation-precision exceeds 0.8. (b) the test set precision at different training + validation TP rates for motifs filtered with precision threshold 0.9 and recall thresholds (Supplementary Table 7) on the training + validation set. In both the training and training + validation sets, eight out of ten highest-frequency implanted ground truth motifs (Supplementary Table 3) are recovered. Note that the seemingly higher precision in the plot for the test set is only an effect of the x-axis starting at a higher value.

To demonstrate our method’s ability to find binding-associated short motifs, we first applied it to a simulated dataset with implanted ground truth motifs of length 3 (Supplementary Table 3). Firstly, all motifs with a maximum length of five and a training set precision greater than 0.9 were searched for. We investigated the validation set precision of these motifs given their number of TP in the training set to determine a recall threshold that ensures generalizability. For rare motifs (low TP), the validation set precision score varies substantially, while more frequent motifs tend to generalize better to the validation set as shown by an increasing precision score (Fig. 2a for motifs of length 3, Supplementary Fig. 6 for motifs of other lengths). For each individual motif length, a training-TP threshold was determined at the point where the smoothed combined validation set precision exceeds 0.8 (Supplementary Table 7). The training-TP thresholds were converted to recall thresholds. Subsequently, the training and validation sets were combined in order to learn a new set of motifs with a precision greater than 0.9 and recall greater than the learned thresholds (Supplementary Table 7). These motifs indeed retain a high smoothed combined precision (>0.8) on the independent test set (Fig. 2b for motifs of length 3, Supplementary Fig. 6 for motifs of other lengths).

Figure 2 furthermore demonstrates that while most ground truth motifs were recovered by our method, this is only possible for ground truth motifs that appear with sufficient abundance, as two out of 10 ground truth motifs were too rare to exceed the recall threshold. Moreover, 19 475 additional motifs were learned on the combined training and validation set. However, this can be explained by the fact that many of these motifs are highly overlapping with the ground truth motifs (Supplementary Fig. 7). Indeed, all these motifs overlap in at least one position with any ground truth motif.

We furthermore applied our method to a second simulated dataset without any ground truth motifs, where binder and non-binder sequences are drawn from the same distribution. In this dataset, there exist no motifs that are truly generalizable to the validation set, and as a consequence the combined validation set precision remains around 0.3 (the fraction of binders) for any number of training-TP (Supplementary Fig. 8), thereby showing that our method is not prone to capturing ‘noise’ motifs if no ground truth motifs are present.

### Discovering short antigen binding-associated motifs in experimental data

**Figure 3:**
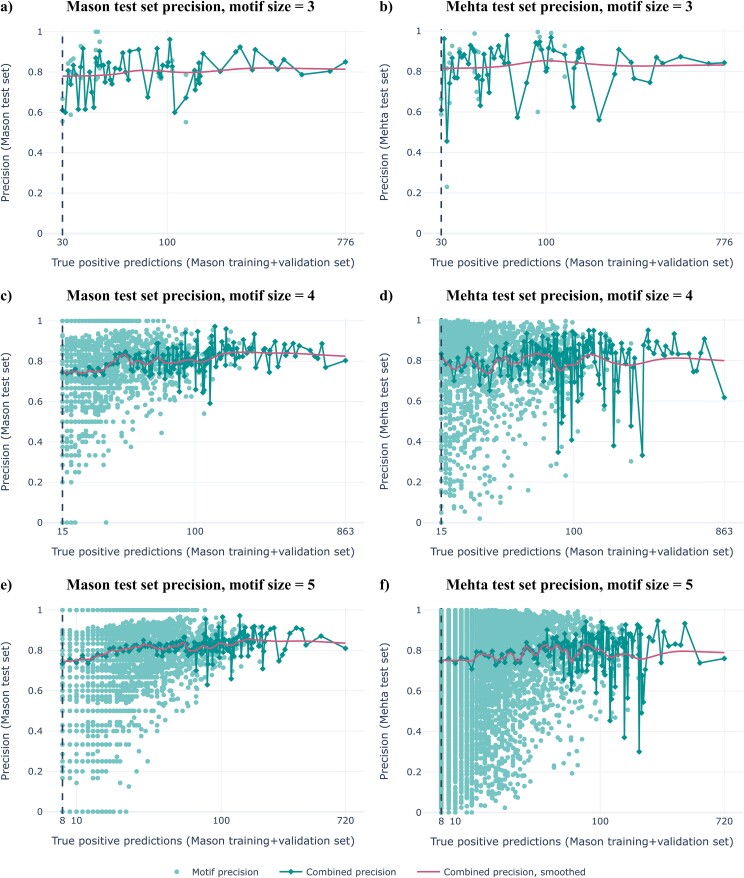
Motifs learned with training precision threshold 0.8 generalize well to unseen data. The figure shows the test set performance of motifs of sizes 3, 4, 5, on the 25% Mason test set (a, c, e) and on the independently generated Mehta set (b, d, f). These motifs were learned using only the Mason training + validation set, yet retain a high smoothed combined precision on the Mehta set. The test set performance of motifs with training precision threshold 0.9 is shown in Supplementary Fig. 9.

We applied our method for finding high precision and recall motifs to a previously published dataset of HER2 binders and non-binders by Mason et al. [32], using training set precision thresholds 0.8 and 0.9, and found 11 336 and 792 motifs respectively (Supplementary Table 8). While using training precision threshold 0.9 yielded motifs with a slightly higher combined precision on the validation set, this difference was only very small, and the use of precision threshold 0.8 allows to retain a far larger number of motifs due to (i) more motifs remaining after filtering the training set with a precision threshold and (ii) allowing a lower recall threshold (Supplementary Fig. 9). The combined precision of the learned motifs remained high on the independent test data, both for training precision thresholds 0.8 (Fig. 3) and 0.9 (Supplementary Fig. 10), showing that the learned motifs are highly generalizable even across the independently generated experimental Mehta dataset.

The motifs learned with training precision threshold 0.8 ranged between 3–5 amino acids in size, whereas with threshold 0.9, they consisted of 4–5 amino acids. Although some positions were favored, the collection of motifs spanned across any position in the 10 amino acid-long sequence (Supplementary Fig. 11). Rather than being locally concentrated, the motifs had a tendency to contain multiple small gaps and thus be spread out across the CDR3 sequence (Supplementary Fig. 12). The set of binder sequences containing each individual motif showed a high amount of variability across the non-motif positions (Supplementary Fig. 13), indicating that the learned motifs are not simply capturing groups of binder sequences with high overall sequence similarity. A clustering analysis of the 792 motifs found with threshold 0.9 showed high variability among the total set of motifs, with some subsets of highly similar motifs but no clear outliers (Supplementary Fig. 14).

The preferential use of certain positional amino acids can largely be explained by the relative difference in the amino acid presence in positive and negative classes (Supplementary Fig. 2). However, some of the learned motifs contained amino acids that in the marginal distributions preferentially occur in the negative class, such as F1, V3, L3, A5, S5, F7, and L9 (Supplementary Figs. 2 and 11), demonstrating that the specific combination of amino acids contained within these motifs is predictive of antigen binding, rather than just the individual amino acids per se being more abundant in the positive class. In other words, the motifs do not merely capture main effects, but rather a combination of main and interaction effects.

### A simple motif-based classifier performs comparably to deep learning, and generalizes better to independent experimental data

**Figure 4:**
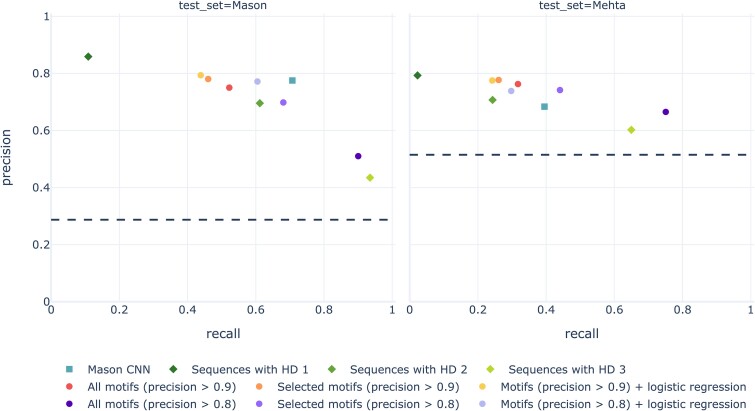
Motif-based classifiers may perform comparable to the CNN, in particular on the independent test set. While the Mason CNN has the best tradeoff between precision and recall on the 25% Mason test set, it shows worse generalisability to the independent Mehta test set than the selected motifs classifier with precision threshold 0.8. The exact precision and recall scores of each model, as well as the accuracy and balanced accuracy can be found in Supplementary Table 9. The horizontal line represents the proportion of positive sequences in the dataset, i.e., the precision of a no-skill classifier.

We compared the performance of six motif-based classifiers, three Hamming distance-based classifiers and the original CNN proposed by Mason et al. in the paper introducing the Mason dataset [32] (Fig. 4, Supplementary Table 9) after training using the Mason training and validation sets. While the CNN marginally stands out in both precision and recall on the Mason test set, its performance on the independent Mehta test set is pareto-dominated by the *selected motifs* classifier with training precision threshold 0.8. This far simpler motif-based model had many (correct) predictions in common with the CNN, although the type of misclassifications were not notably similar between the two models (Supplementary Tables 10 and 11). The reduced collections of motifs learned by the *selected motifs* classifiers contained just 178 out of 11,336 motifs learned with training precision threshold 0.8, and 229 out of 792 motifs learned with threshold 0.9 (Supplementary Fig. 15). Furthermore, *all motifs* with training precision threshold 0.8 outperformed any other model on the Mehta test set in terms of accuracy and balanced accuracy, due to its high recall (Supplementary Table 9). The Hamming distance-based classifiers had a relatively poor precision-recall tradeoff compared to all other models. Furthermore, gapped motifs learned with a 0.9 threshold showed better generalizability to the Mehta dataset than contiguous k-mers selected based on highest frequency when used as features for a logistic regression model (Supplementary Table 12).

## Discussion

While several studies have been conducted under the assumption that the presence of a short motif in the AIR CDR3 is the driving factor for its antigen binding status [14–17, 26, 27], this hypothesis has not been investigated directly. In this work, we have shown that there indeed exist motifs composed of a few amino acids in fixed positions of the antibody CDRH3, whose presence is nearly sufficient to predict antigen binding in a mutagenesis dataset where other variable regions were kept the same. We found several such motifs that were highly predictive of antigen binding on a 25% hold-out test set, and even on an independently generated experimental replicate (Fig. 3). Importantly, these motifs were learned using sets of otherwise highly variable sequences (Supplementary Fig. 2), indicating that the presence of the motif itself, rather than overall high sequence similarity, is the determining factor for antigen binding in these datasets. We furthermore showed that the motifs identified by our method can be used as components for interpretable ML methods, resulting in an ML classifier that despite its simplicity outperformed the CNN architecture proposed by Mason et al. [32] on the independent Mehta test dataset (Fig. 4).

Previous studies relying on short CDR3 motifs have limited their scope to local motifs with no or few gaps [13–17, 26], as this corresponds to the typical shape of paratopes [3]. In contrast, the antigen binding associated motifs we observed were most often highly dispersed, containing multiple gaps, and only a small fraction of the motifs were contiguous (Supplementary Figs. 12 and 14). The 3D structure of the Trastuzumab Fab fragment complexed with HER2 has been experimentally determined [48] (Supplementary Fig. 16). Investigations of this structure have shown that the closest contact residues are 99 W, 103G, and 105Y [49], while residues 102D and 104F have additionally been reported as contact residues [32]. These residues correspond to respective positions 1, 5, 7 and 4, 6 in our numbering. The most commonly used positions in the antigen-binding associated motifs were instead 5, 8, and 9, while position 4 was least common (Supplementary Figs. 11 and 16). This indicates that the paratope information for one particular antibody, which informs about positions important for physical binding, does not necessarily inform about which positions are statistically important for binding in a set of related variants with possible structural variations.

The currently used mutagenesis datasets only contain information about specificity to a single target antigen. As dense sequence data for a larger variety of antigens becomes available, the described method can be applied to investigate how the characteristics of antigen binding motifs may overlap or differ across binding targets. Furthermore, the positional amino acid distribution in the Mason dataset was by design skewed to contain larger proportions of amino acids found to be favorable for binding when considered in isolation [32]. While this skewing was necessary to create a large number of binder examples, it also resulted in loss of amino acid variability in certain positions, most notably position 7 (Supplementary Fig. 2). The implications of the data generating process for the learning of antigen binding-associated motifs remains to be investigated.

Although the precision of the learned motifs on the Mason dataset appears to be comparable to the performance on the Mehta test set (Fig. 3), it should be noted that the Mehta test set is more balanced (Supplementary Table 1), making it theoretically easier for a motif to score a higher precision on the Mehta set due to the higher fraction of TPs. We did however not observe a substantial increase in precision for motifs on the Mehta set, possibly due to distribution differences between the Mason and Mehta datasets (Supplementary Fig. 4), which are within the range of distribution differences observed between binders and non-binders in the Mason and Mehta datasets (Supplementary Figs. 2 and 3). In this work we have considered training precision thresholds 0.8 and 0.9, and subtracted 0.1 to find the respective validation set precision thresholds which were used as a guide for setting the recall threshold. Given the fact that the expected precision of any motif depends directly on the class balance, the relationship between class balance and optimal threshold setting techniques should be explored further, and may help improve motif generalizability for a variety of datasets.

Another future direction of our work could be to investigate how the ability to predict antigen binding would change if different definitions and parameterizations of a ‘short motif’ were used. We here investigated motifs consisting of amino acids at specific positions (with possible gaps in between), given the context of a fixed CDRH3 length, heavy chain V/J genes, and light chain. This could be contrasted with motifs that are position-independent, contain flexible gap sizes, allow synonymous amino acids within motifs by grouping together amino acids with similar (biophysicochemical) properties, and have fixed or flexible definitions of the other variable regions. By comparing the classification performance of (sets of) motifs derived using different parameterizations, we could get a deeper insight into the dimensions across which antigen binding ability can most clearly be separated. However, computational limitations could pose a bottleneck for the investigation of certain motif parameterizations. To make the current study computationally feasible, we relied on algorithms that reduce the motif search space, vectorized comparison operations, parallelization and intermediate result caching. In order to investigate an even larger motif search space, sophisticated algorithmic solutions or heuristics need to be employed.

In conclusion, we here used large-scale mutagenesis data to directly investigate a central yet unresolved question in the AIR field: whether antigen binding status is (to some degree) driven by the presence of short motifs in the immune receptor sequence. This was operationalized as investigating whether there exists short motifs sufficient for predicting antigen binding status. A set of such motifs were indeed found in the considered dataset, and when combined in a simple classification algorithm, they even outperformed a state-of-the-art deep learning model in generalization to an independently generated dataset. We have made our methodology available as a framework that can be used to gain further insights into which factors are driving immune receptor binding as further large-scale datasets on antibody and TCR binding become available in the future.

**Key Points**

- We present a novel method for the discovery of antigen binding-associated motifs in the CDR3 sequences of antibodies and t-cell receptors.
- We use an experimental antibody CDR3 dataset to find a set of HER2 binding-associated motifs, which are shown to have high generalizability to an independently generated experimental replicate dataset.
- We create a simple motif-based classifier which shows comparable performance to the deep learning model originally proposed for the antibody CDR3 dataset, and outperforms this deep learning model on the experimental replicate dataset.

## Supplementary Material

final_scheffer_short_motif_supplementary_bbae537

## Data Availability

The datasets analysed in this article have been previously published [32, 42]. The source code underlying this article has been integrated into the open source AIRR-ML platform immuneML [20] (version 3.0.0a4) and is available on Figshare https://doi.org/10.6084/m9.figshare.25712421. A tutorial for the discovery of positional motifs using precision and recall thresholds with immuneML can be found at: https://docs.immuneml.uio.no/latest/tutorials/discover_motifs_precision_recall.html. Additional scripts for data preprocessing, simulation, and parsing of the immuneML results, as well as immuneML analysis specifications have been deposited on Figshare https://doi.org/10.6084/m9.figshare.25664778.v1. Furthermore, the intermediate immuneML results and raw data underlying the manuscript figures have been deposited to Zenodo: https://doi.org/10.5281/zenodo.10047590.

## Notes

Present Address: Lonneke Scheffer, La Jolla Institute for Immunology, La Jolla, CA, United States. Eric Emanuel Reber, Department of Biosystems Science and Engineering, ETH Zürich, Zürich, Switzerland.

Maria Chernigovskaya, Department of Microbiology, Tumor and Cell Biology, Karolinska Institute, Solna, Sweden.

Rahmad Akbar, Novo Nordisk, Denmark.
