# Supplementary material for: Predictability of antigen binding based on short motifs in the antibody CDRH3
Source: Brief Bioinform. 2024 Oct 22;25(6):bbae537. doi: 10.1093/bib/bbae537 (PMC11495870; doi:10.1093/bib/bbae537)
Supplement: final_scheffer_short_motif_supplementary_bbae537 [file final_scheffer_short_motif_supplementary_bbae537.docx]

# Supplementary materials

**
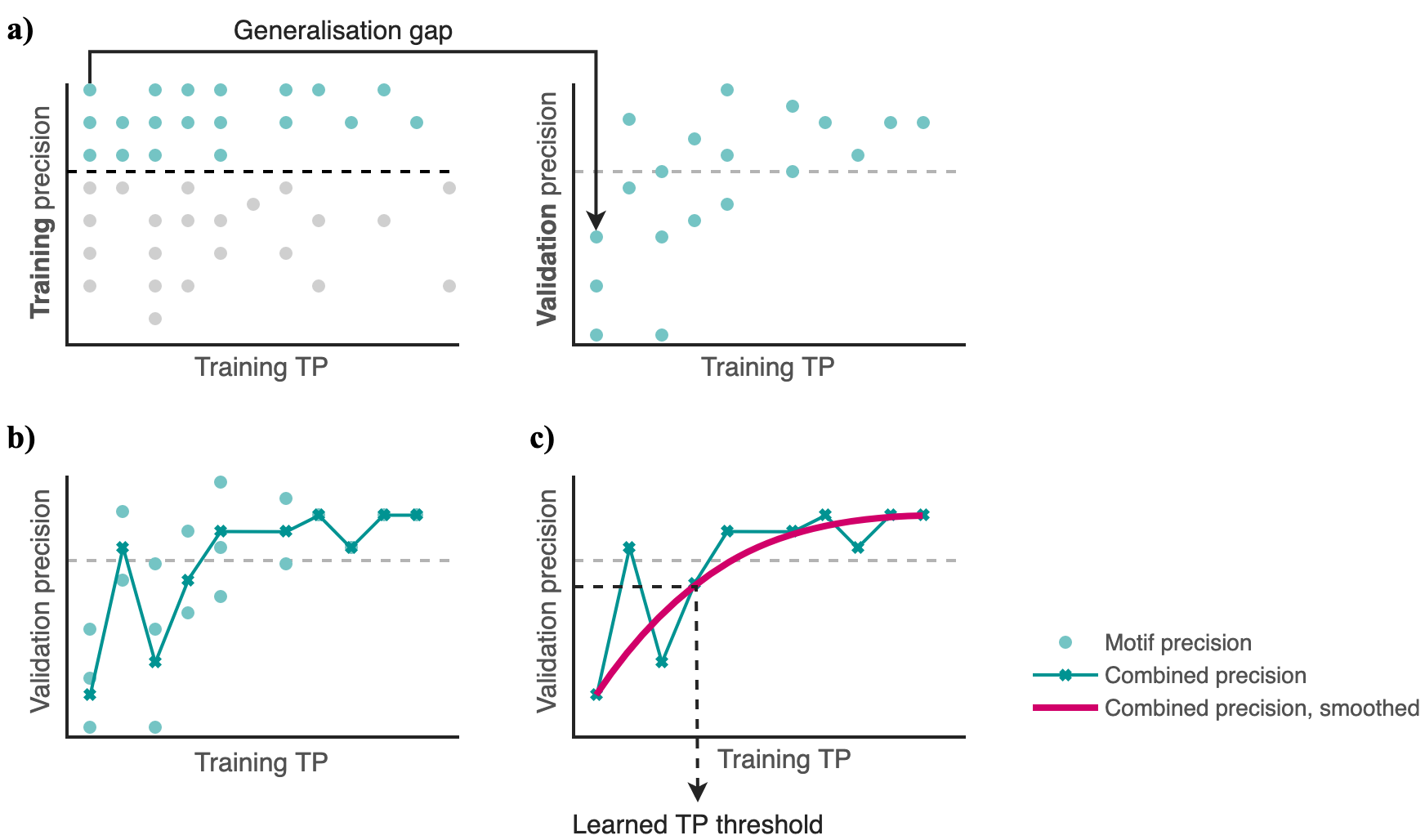
**

**Supplementary Figure 1: The steps to compute the optimal TP threshold given a training precision threshold.** After all motifs with a TP count of at least 2 have been discovered, (a) the motifs with a precision on the training set below the given threshold (e.g., 0.8 or 0.9) are discarded. The generalisation gap is the difference between the precision on the training set and the previously unseen validation set. This generalisation gap is expected to be larger for rare motifs (low TP). (b) The combined validation set precision is computed for each group of motifs that has the same number of TP occurrences on the training set. (c) The combined validation precision curve is smoothed, and the training TP threshold is determined to be at the point where this smoothed curve crosses the training precision threshold – 0.1. The recall threshold is equal to the TP threshold is divided by the total number of binders in the training set.

**Supplementary Table 1: The number of sequences left in the Mason and Mehta datasets after preprocessing.**

|  | Mason dataset | Mehta dataset |
| --- | --- | --- |
| Number of binders | 10,310 | 172,149 |
| Number of non-binders | 25,579 | 162,186 |
| Total number of sequences | 35,889 | 334,335 |

**Supplementary Table 2: The number of overlapping sequences between the Mason and Mehta datasets.**

|  | Mehta binders | Mehta non-binders |
| --- | --- | --- |
| Mason binders | 98 | 22 |
| Mason non-binders | 14 | 7 |


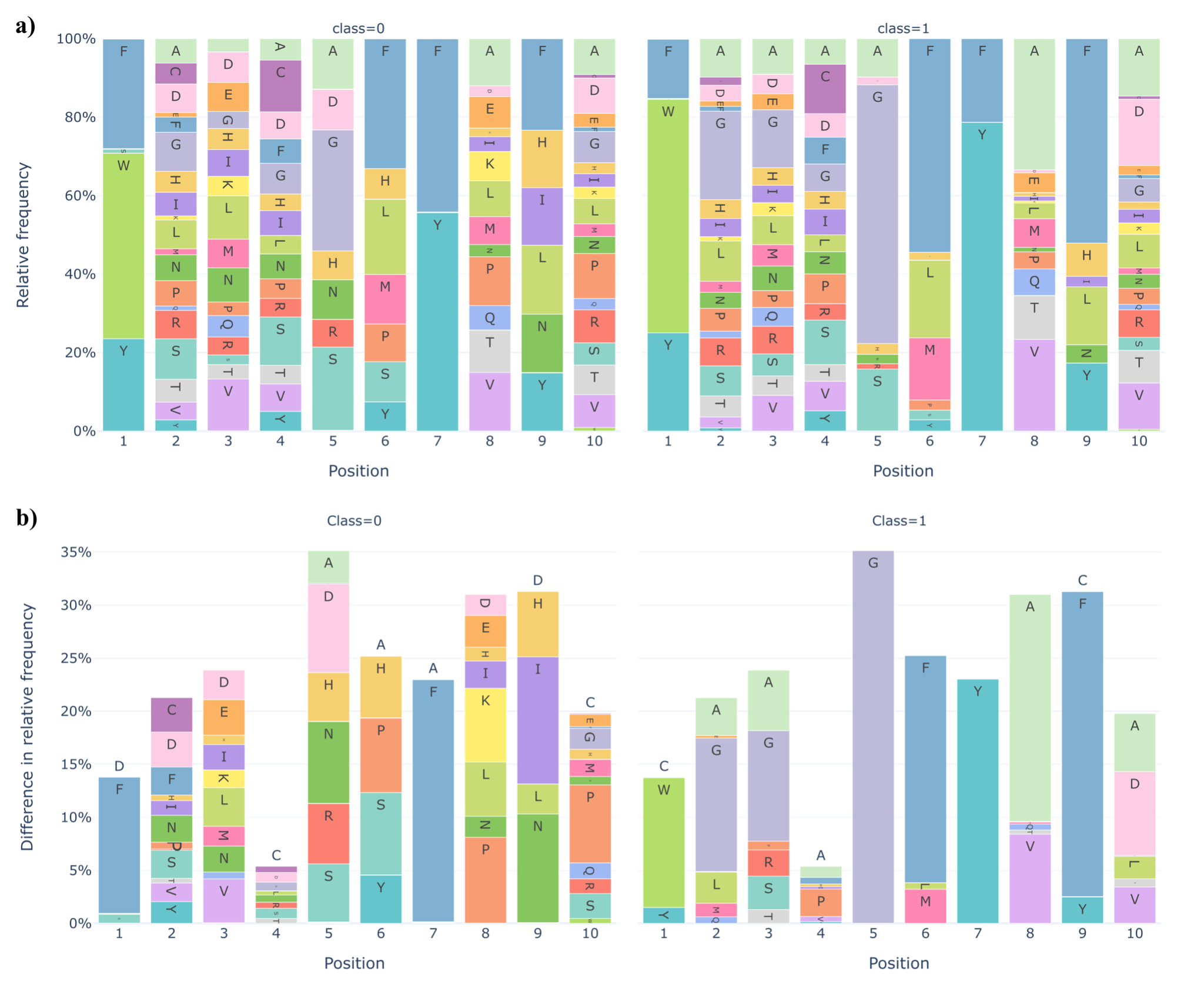


**Supplementary Figure 2: The positional amino acid distribution of the Mason dataset.** Slight differences can be observed in the amino acid usage of the positive (class=1) and negative (class=0) sequences in the Mason dataset. (a) The overall positional distributions of the sequences. (b) The overrepresentation of individual positional amino acids in the positive and negative classes. Positions 1–10 as shown in this figure correspond to IMGT CDRH3 positions 99–108.

**
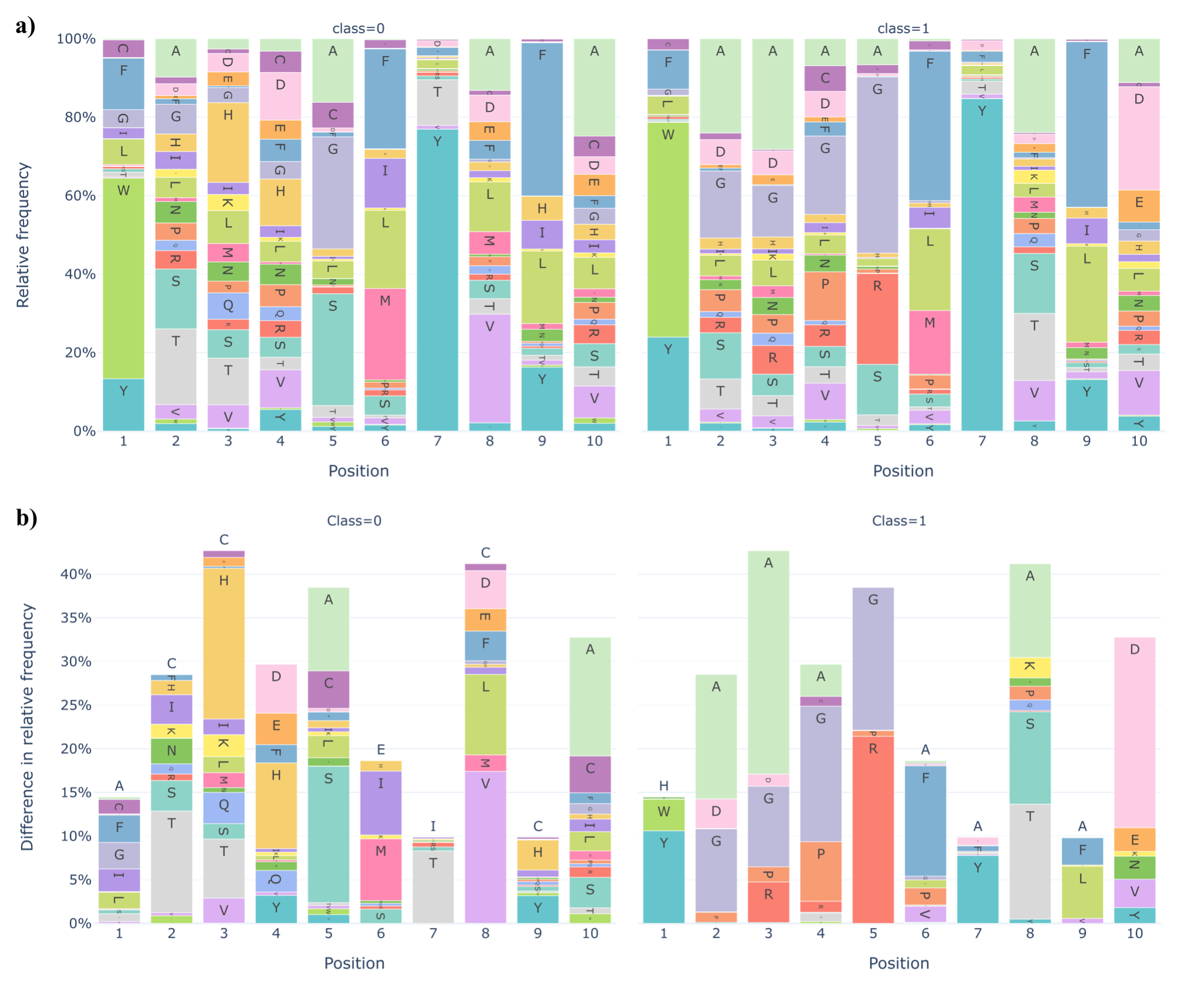
**

**Supplementary Figure 3: The positional amino acid distribution of the Mehta dataset.** (a) The overall positional distributions of the sequences. (b) The overrepresentation of individual positional amino acids in the positive and negative classes. While the experimental protocol was matched to that of the Mason dataset, the resulting distributions of the preprocessed Mason (Supplementary Figure 2) and Mehta datasets do display differences (Supplementary Figure 4). Positions 1–10 as shown in this figure correspond to IMGT CDRH3 positions 99–108.

**
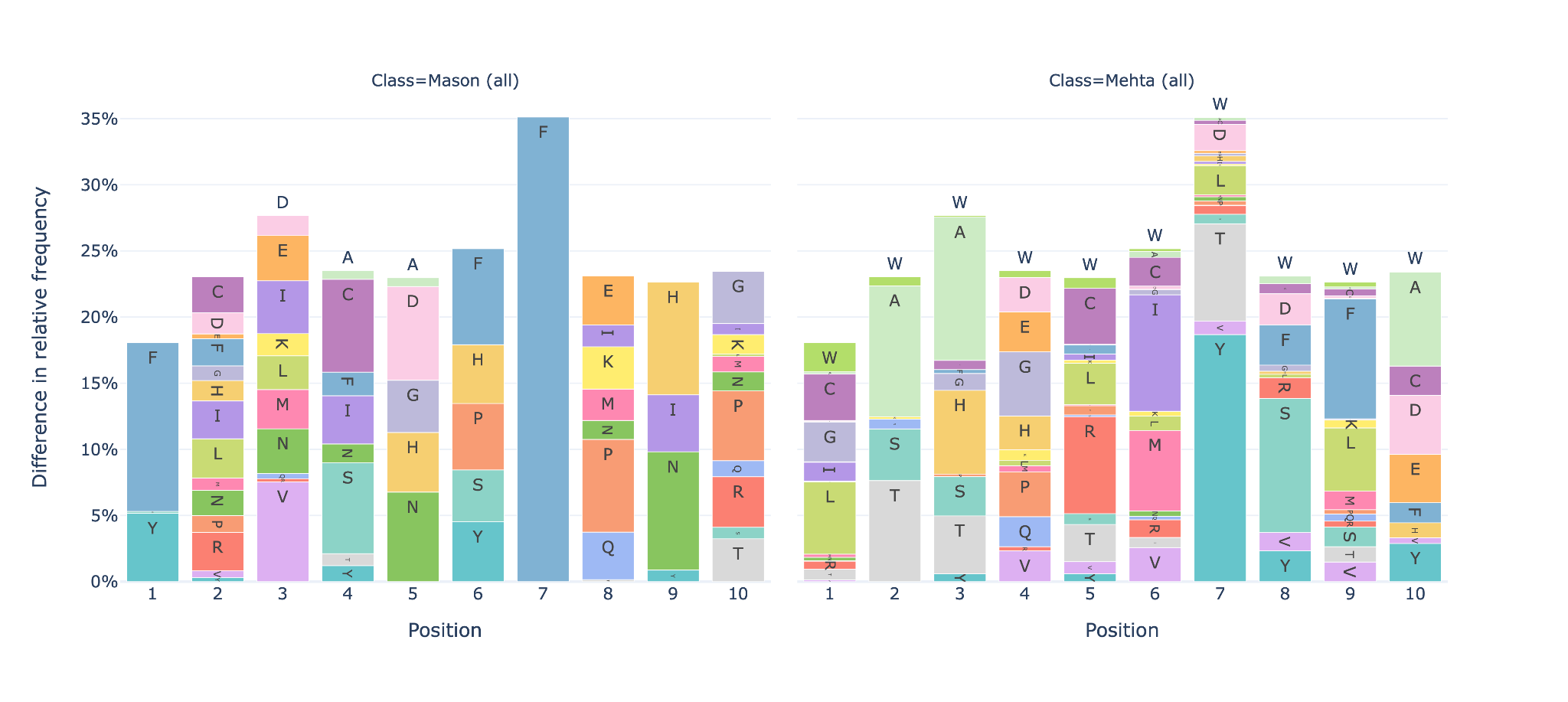
**

**Supplementary Figure 4: The relative difference between the positional amino acid distribution of the Mason and Mehta datasets.** Despite the fact that the Mehta dataset was generated based on the same positional weights defined by Mason et al. [32], differences can be found in the distributions of the two datasets. A higher diversity of amino acids is observed across the Mehta dataset in positions 1, 6, 7, 8 and 9, while the Mason dataset is more diverse in position 2. A more detailed breakdown of the individual distributions of the Mason and Mehta datasets are shown in Supplementary Figures 2 and 3 respectively. Positions 1–10 as shown in this figure correspond to IMGT CDRH3 positions 99–108.

**
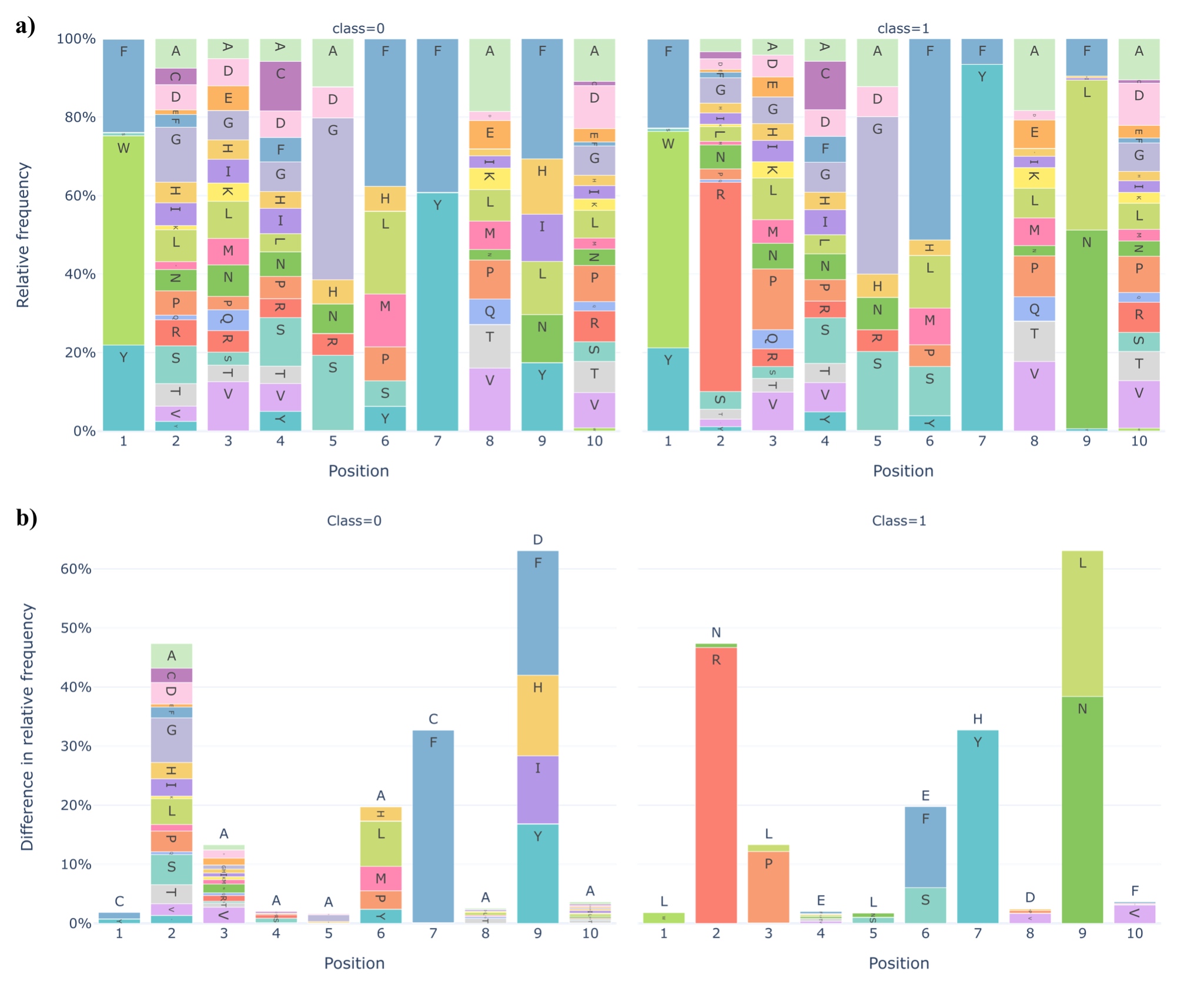
Supplementary Figure 5: The positional amino acid frequency of the simulated dataset.** The introduction of ground truth motifs in the binder sequences causes shifts in the positional amino acid frequency distribution of the positive classes compared to the negative classes. However, slight differences in the positional frequency distribution of binder and non-binder sequences can also be observed in the Mason dataset (Supplementary Figure 2). (a) The overall positional distributions of the sequences. (b) The overrepresentation of individual positional amino acids in the positive and negative classes. Similarly to the Mason data distribution, higher variability is observed among the amino acids overrepresented in the negative class.

**Supplementary Table 3: The binding-associated ground truth motifs and their implanting rates in the synthetic dataset.** Note that while groundtruth motifs were explicitly excluded from non-binder sequences, some binder sequences may by chance contain multiple groundtruth motifs. Therefore, the values in the column named ‘Number of binder sequences’ sum to a higher total value (10.478) than the actual number of binder sequences (10.000).

| Motif | Number of binder sequences | In the training set | In the validation set | In the test set |
| --- | --- | --- | --- | --- |
| G5, P8, I9 | 10 | 7 | 1 | 2 |
| Y1, M6, F7 | 24 | 8 | 6 | 10 |
| Y1, V8, F9 | 65 | 29 | 14 | 22 |
| S5, S6, F7 | 88 | 51 | 16 | 21 |
| F1, L3, F9 | 169 | 79 | 42 | 48 |
| N2, Y7, V10 | 332 | 165 | 85 | 82 |
| S6, Y7, F9 | 644 | 318 | 159 | 167 |
| W1, P3, L9 | 1,303 | 640 | 329 | 334 |
| F6, Y7, L9 | 2,838 | 1,409 | 730 | 699 |
| R2, Y7, N9 | 5,005 | 2,478 | 1,226 | 1,301 |

**Supplementary Table 4: The immuneML encoding and ML method classes that were used to represent each of the classifiers.** All immuneML classes that were newly implemented to support this work are marked with an asterisk.

|  | immuneML encoding | immuneML ML method |
| --- | --- | --- |
| All motifs classifier | Motif* | BinaryFeatureClassifier* (keep_all=True) |
| Selected motifs classifier | Motif* | BinaryFeatureClassifier* (keep_all=False, patience=5) |
| Motifs + logistic regression | Motif* | LogisticRegression |
| Hamming distance classifier | SimilarToPositiveSequence* | BinaryFeatureClassifier* (keep_all=True) |
| Mason CNN | OneHot | KerasSequenceCNN* |

**Supplementary Table 5: The set of hyperparameters considered when training logistic regression models.** The scikit-learn implementation of logistic regression was used. For any hyperparameter not shown in this table, the scikit-learn default value was used. The optimal selected values for the hyperparameters ‘C’ and ‘penalty’ are marked with an asterisk. These hyperparameters were optimal for both logistic regression models using the motifs found with precision thresholds 0.8 and 0.9.

| Hyperparameter | Value(s) |
| --- | --- |
| C | 0.01, 0.1, 1*, 10, 100 |
| penalty | l1, l2* |
| max_iter | 1,000 |
| solver | saga |

**Supplementary Table 6: The set of hyperparameters used for the CNN.** These hyperparameters match the optimal hyperparameters found in the original study by Mason et al. [32]

| Hyperparameter | Value(s) |
| --- | --- |
| Batch size | 16 |
| Convolutional layer: Number of filters | 400 |
| Convolutional layer: kernel size | 5 |
| Convolutional layer: stride | 1 |
| Convolutional layer: activation function | ReLU |
| Dropout layer: dropout rate | 0.2 |
| Max pool layer: pool size | 2 |
| Max pool layer: stride | 1 |
| Dense layer: number of nodes | 300 |
| Dense layer: activation function | ReLU |

**
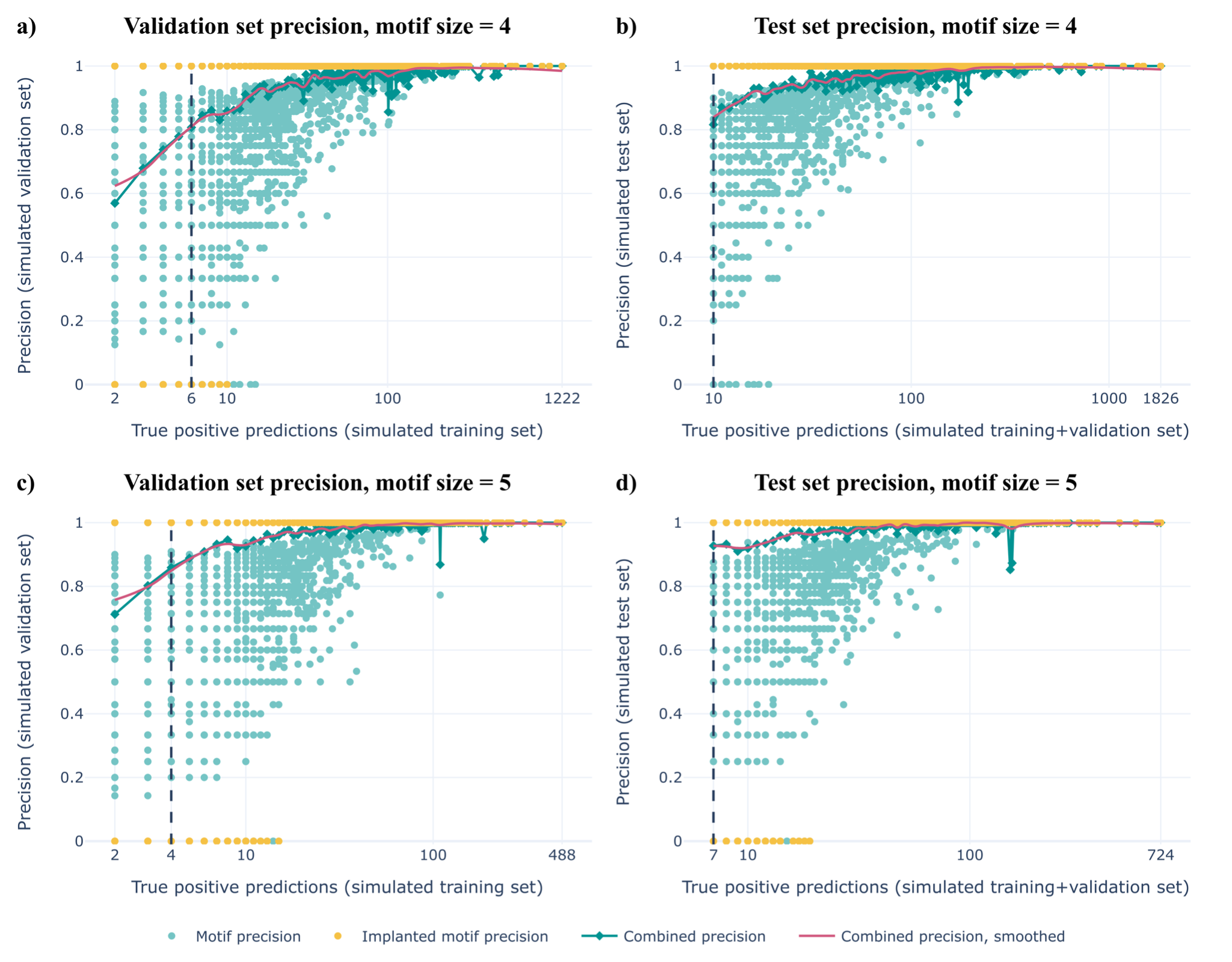
**

**Supplementary Figure 6: Performance of learned motifs of various sizes on the simulated datasets.** The ground truth implanted motifs of length 3 are shown in Figure 2. This figure shows the performance of motifs of size 4 (a) validation set and (b) test set, and size 5 (c) validation set and (d) test set. When applying the recall thresholds determined on the validation sets, the motifs learned on the training + validation set retain a high combined precision on the test set. Motifs containing any of the ground truth implanted motifs (Supplementary Table 3) are marked in yellow.

**Supplementary Table 7: Precision and recall thresholds and subsequent numbers of motifs found in the training and training + validation sets for the simulated dataset.** The training TP threshold is set at the point where the combined validation precision exceeds the precision threshold – 0.1, i.e., 0.8. The total number of possible motifs represents the number of combinations of positional amino acids that were observed at least once in the entire Mason dataset (impossible amino acids are thus excluded). The last two columns refer to the number of motifs left after applying the precision and recall and thresholds.

| Motif size | Precision threshold | Training set TP threshold | Recall threshold | Total possible motifs | Motifs found in *training* set | Motifs found in *training + validation* set |
| --- | --- | --- | --- | --- | --- | --- |
| 1 | 0.9 | NA | NA | 185 | 0 | 0 |
| 2 | 0.9 | 2,481 | 0.500908540278619 | 15,383 | 1 | 0 |
| 3 | 0.9 | 14 | 0.002826569755703614 | 757,075 | 243 | 209 |
| 4 | 0.9 | 6 | 0.0012113870381586917 | 24,421,116 | 5,600 | 4,413 |
| 5 | 0.9 | 4 | 0.0008075913587724611 | 539,487,840 | 23,365 | 14,861 |


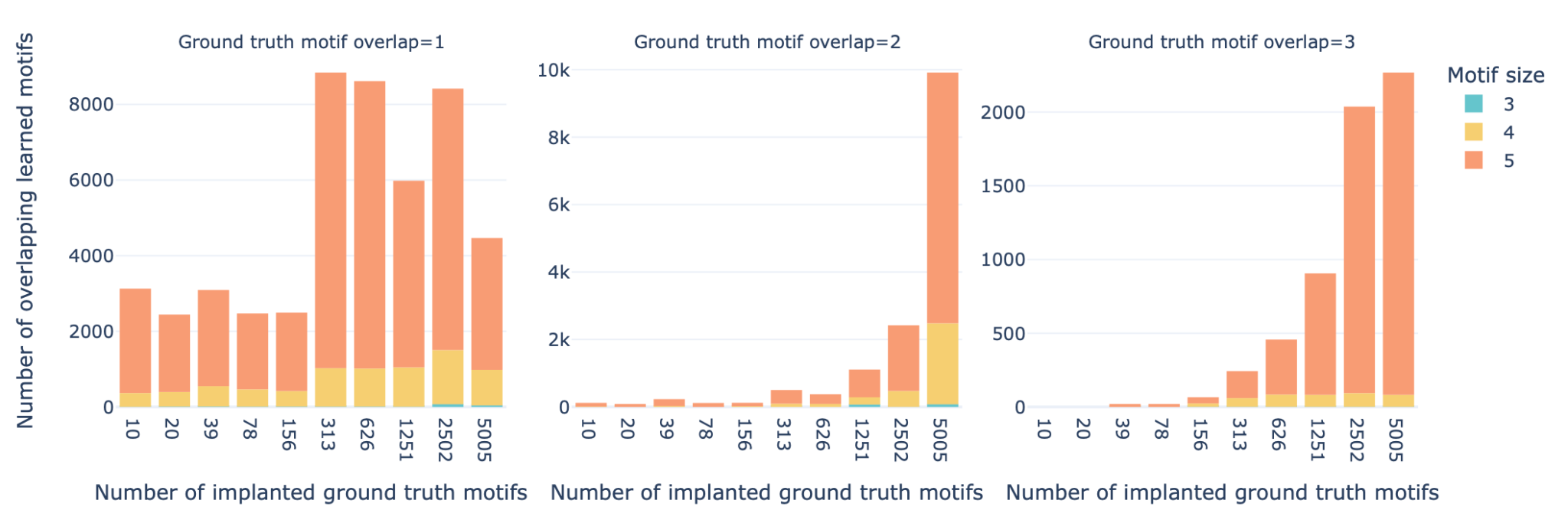


**Supplementary figure 7: Many of the learned set of motifs are partially overlapping with the implanted ground truth motifs.** The ground truth motifs with high implanting rates show a larger number of overlapping learned motifs than those with low implanting rates. Not only is it easier to detect the ground truth motifs with a high implanting rate (Figure 2), but more associated motifs are learned.


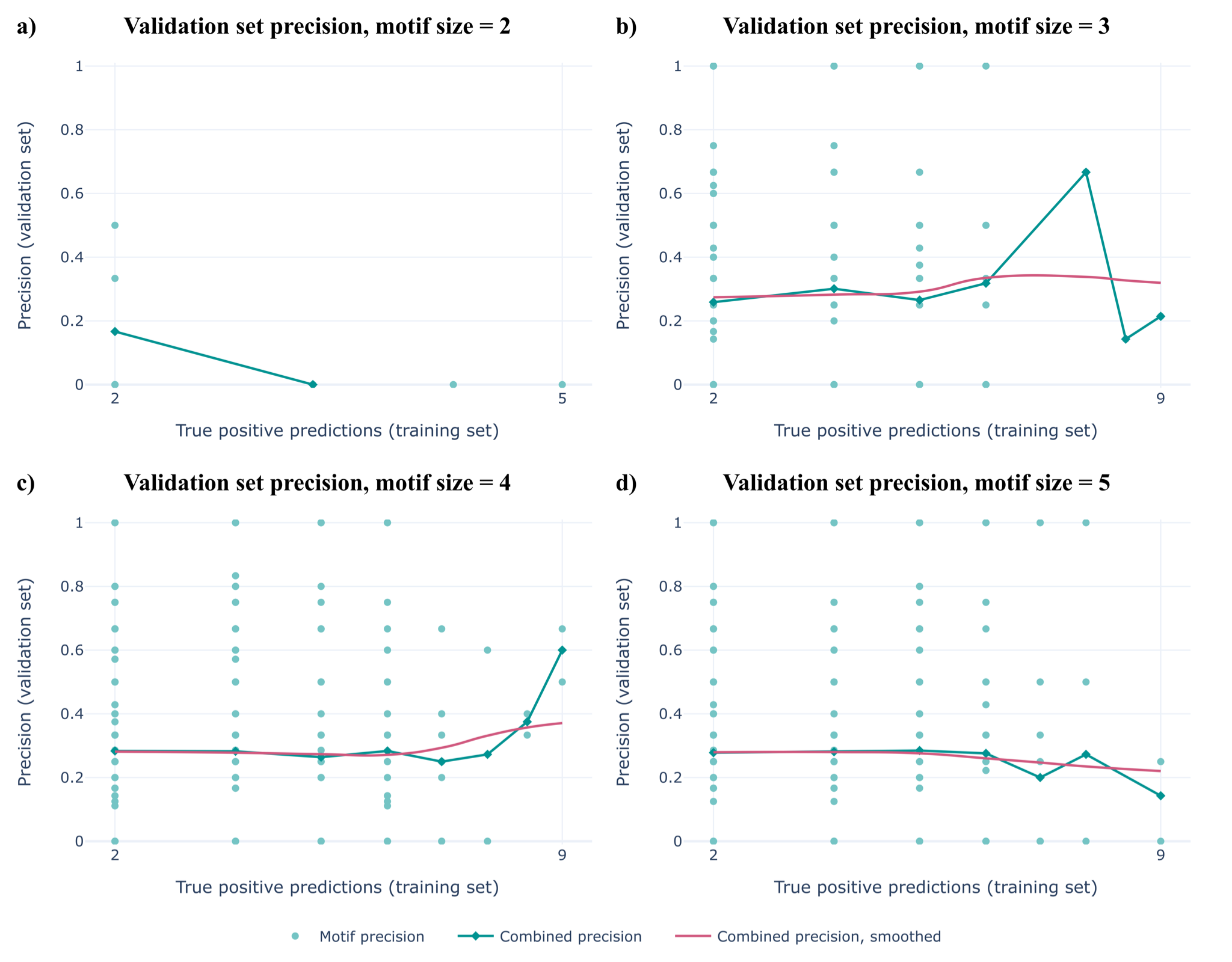


**Supplementary Figure 8: No motifs are learned when using a dataset without implanted ground truth motifs.** The plots show the performance of motifs with a precision > 0.9 on the validation set, for motifs of sizes (a) 2, (b) 3, (c) 4 and (d) 5. When a dataset does not contain ground truth motifs, some individual motifs may have a high precision on both the training and validation set by chance. However, the combined precision of all motifs found with a certain training-TP rate remains around 0.3, which corresponds to the fraction of binders in the dataset.

**Supplementary Table 8: Precision and recall thresholds and subsequent numbers of motifs found in the training and training + validation sets for the Mason dataset.** The training TP threshold is set at the point where the combined validation precision exceeds the precision threshold – 0.1. The total number of possible motifs represents the number of combinations of positional amino acids that were observed at least once in the entire Mason dataset (impossible amino acids are thus excluded). The last two columns refer to the number of motifs left after applying the precision and recall and thresholds.

| Motif size | Precision threshold | Training set TP threshold | Recall threshold | Total possible motifs | Motifs found in *training* set | Motifs found in *training + validation* set |
| --- | --- | --- | --- | --- | --- | --- |
| 1 | 0.8 | NA | NA | 185 | 0 | 0 |
| 2 | 0.8 | NA | NA | 15,383 | 0 | 0 |
| 3 | 0.8 | 20 | 0.003850596842510589 | 757,075 | 104 | 73 |
| 4 | 0.8 | 10 | 0.0019252984212552945 | 24,421,116 | 2,167 | 1,707 |
| 5 | 0.8 | 5 | 0.0009626492106276473 | 539,487,840 | 13,887 | 9,556 |
| 1 | 0.9 | NA | NA | 185 | 0 | 0 |
| 2 | 0.9 | NA | NA | 15,383 | 0 | 0 |
| 3 | 0.9 | NA | NA | 757,075 | 0 | 0 |
| 4 | 0.9 | 18 | 0.0034655371582595304 | 24,421,116 | 148 | 107 |
| 5 | 0.9 | 11 | 0.002117828263380824 | 539,487,840 | 1,091 | 685 |


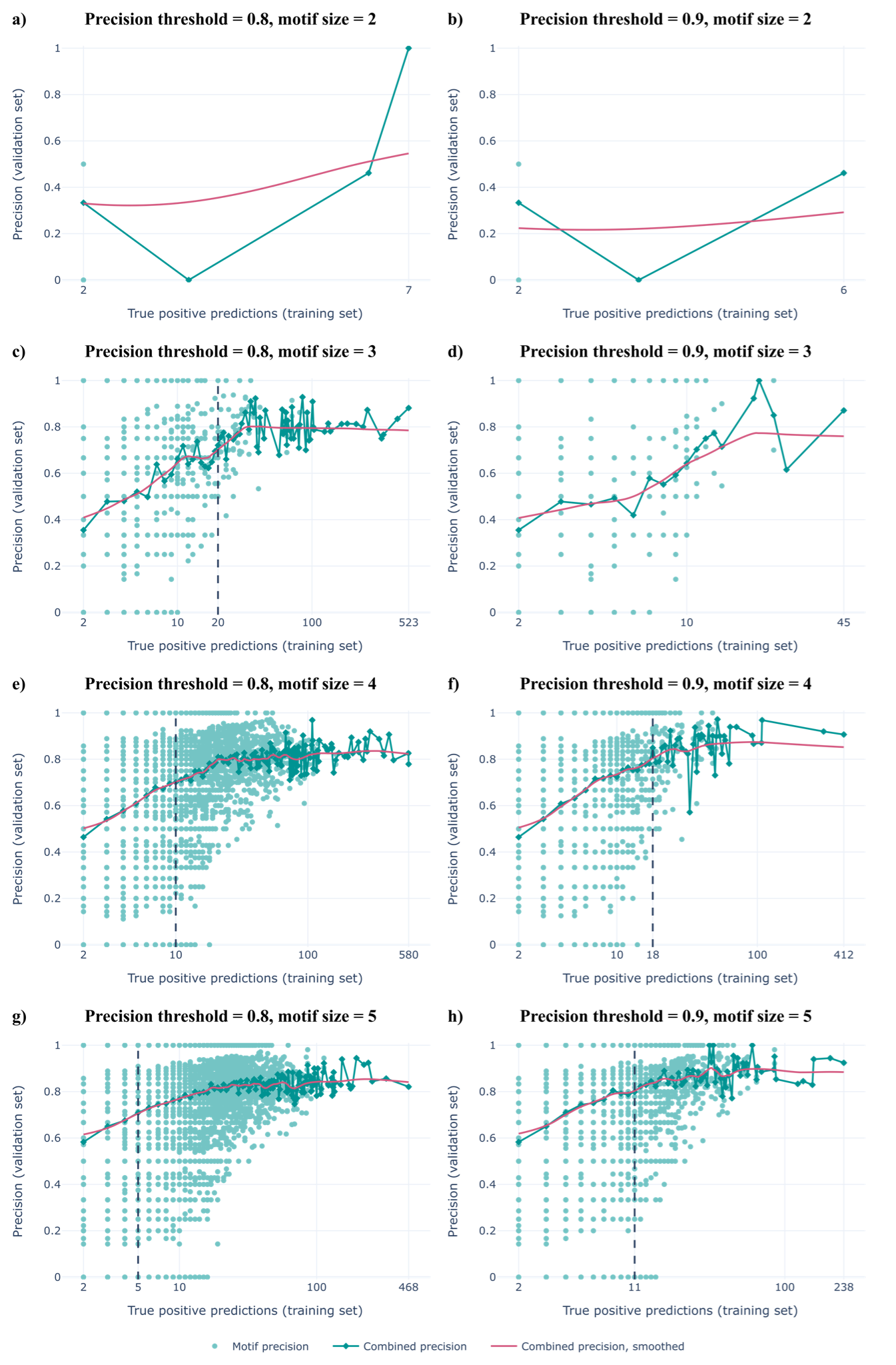


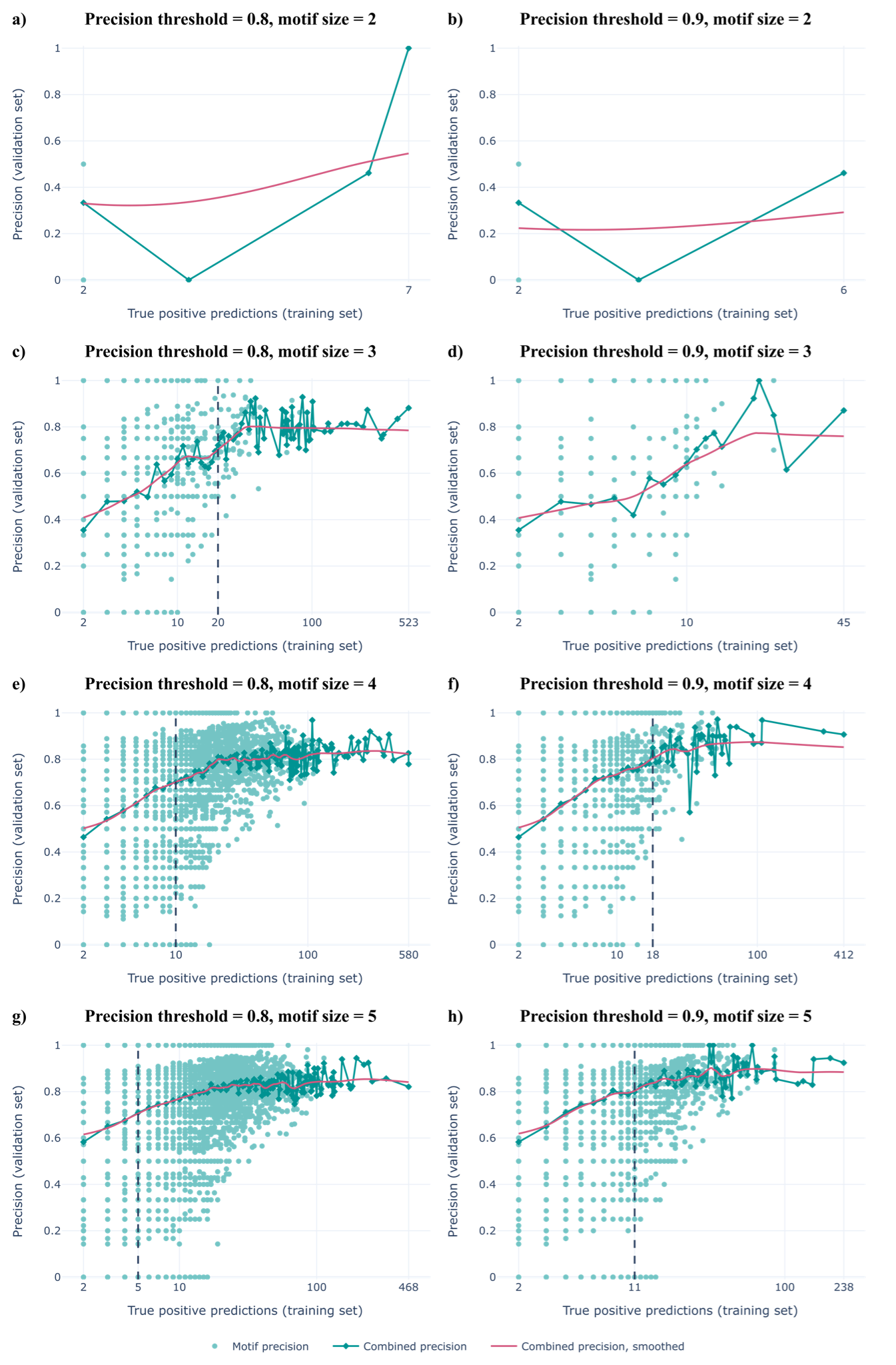


**Supplementary Figure 9: Using training set precision threshold 0.8 as opposed to 0.9 results in more learned motifs, with comparable performance on the validation set.** The motifs of sizes (a, b) 2, (c, d) 3, (e, f) 4, and (g, h) 5 were learned with respective precision thresholds 0.8 or 0.9 on the Mason training set. While there is 0.1 difference between the two training set precision thresholds, the resulting smoothed combined validation precision curves are comparable. Training set TP thresholds (vertical dashed lines) were determined based on the point where the combined precision crosses precision threshold minus 0.1 (0.7 and 0.8 respectively). The corresponding recall thresholds are shown in Supplementary Table 7.

**
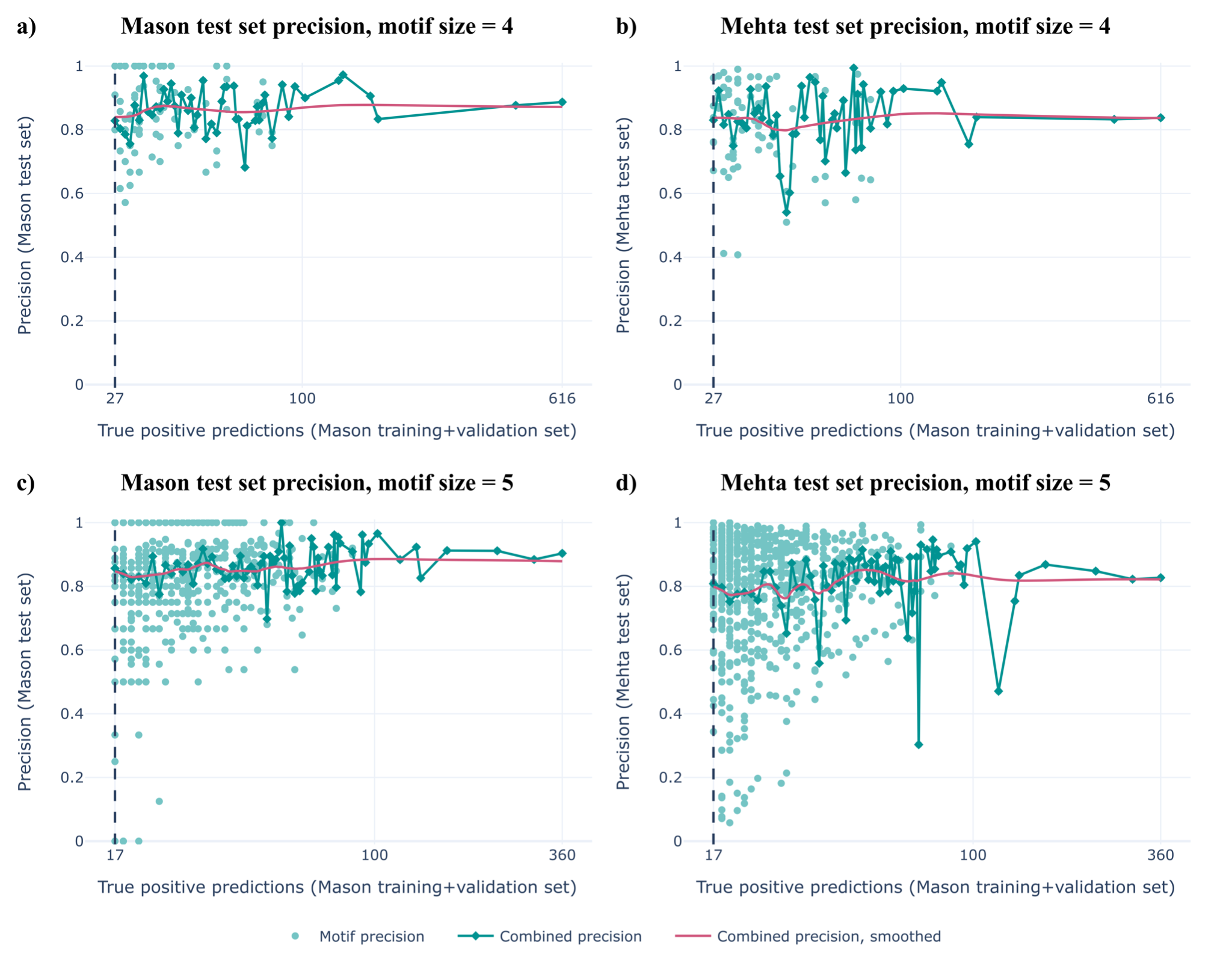
**

**Supplementary Figure 10: Performance of motifs learned with training precision threshold 0.9 on unseen data.** The figure shows the test set performance of motifs of size (a, b) 4 and (c, d) 5 on the Mason test set (25%) and independently generated Mehta set, respectively.


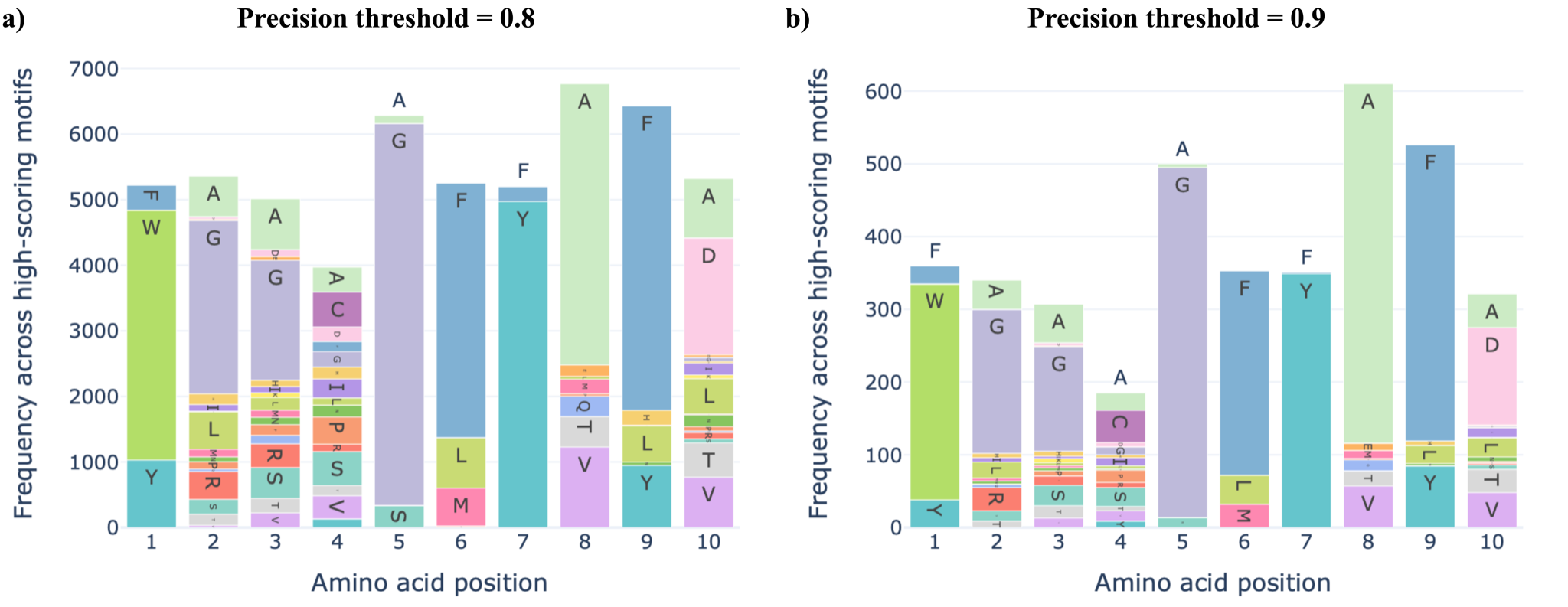


**Supplementary Figure 11: Motifs can be found across all of the 10 variable amino acid positions.** This figure shows how often each amino acid was used within any of the respective 11,336 and 792 motifs learned on the Mason dataset with precision thresholds (a) 0.8 and (b) 0.9. Most of the positional amino acids found across these motifs are overrepresented in the Mason binders (Supplementary Figure 2b), while few motifs contain amino acids that occur more often across non-binders (e.g., F1, S2, V3, A5, S5, F7, and L9). Positions 1–10 as shown in this figure correspond to IMGT CDRH3 positions 99–108.

**
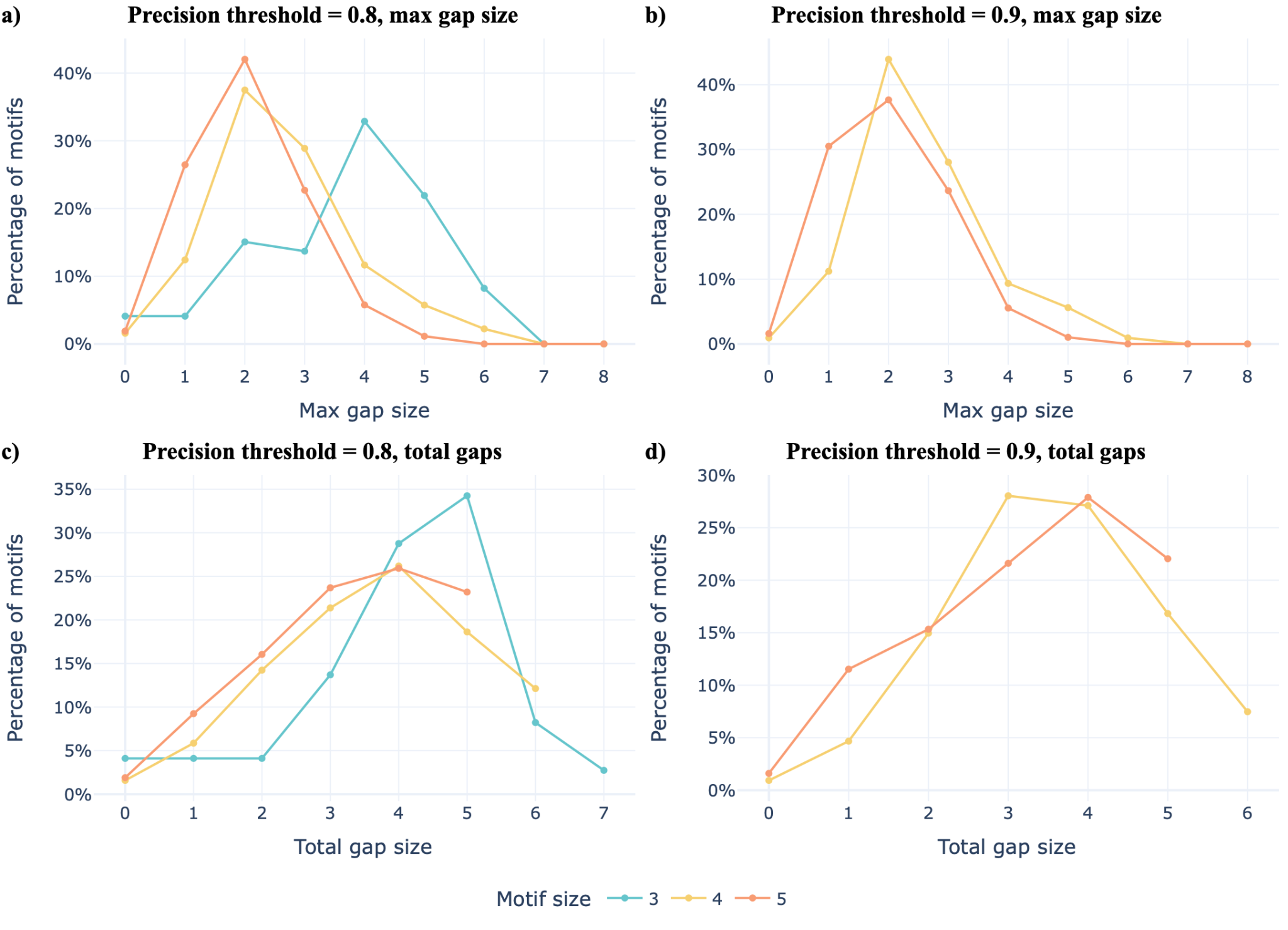
**

**Supplementary Figure 12: Most motifs contain several small gaps.** The figure shows the distribution of (a, b) the maximum gap size in each motif and (c, d) the total number of gap positions within each motif for motifs learned using training precision thresholds 0.8 and 0.9, respectively. Many motifs contain a large number of gap positions, while the maximum gap size is most often only 2 (except motifs of length 3, which contain longer gaps), meaning these motifs are spread out across the sequence rather than being locally concentrated.

**
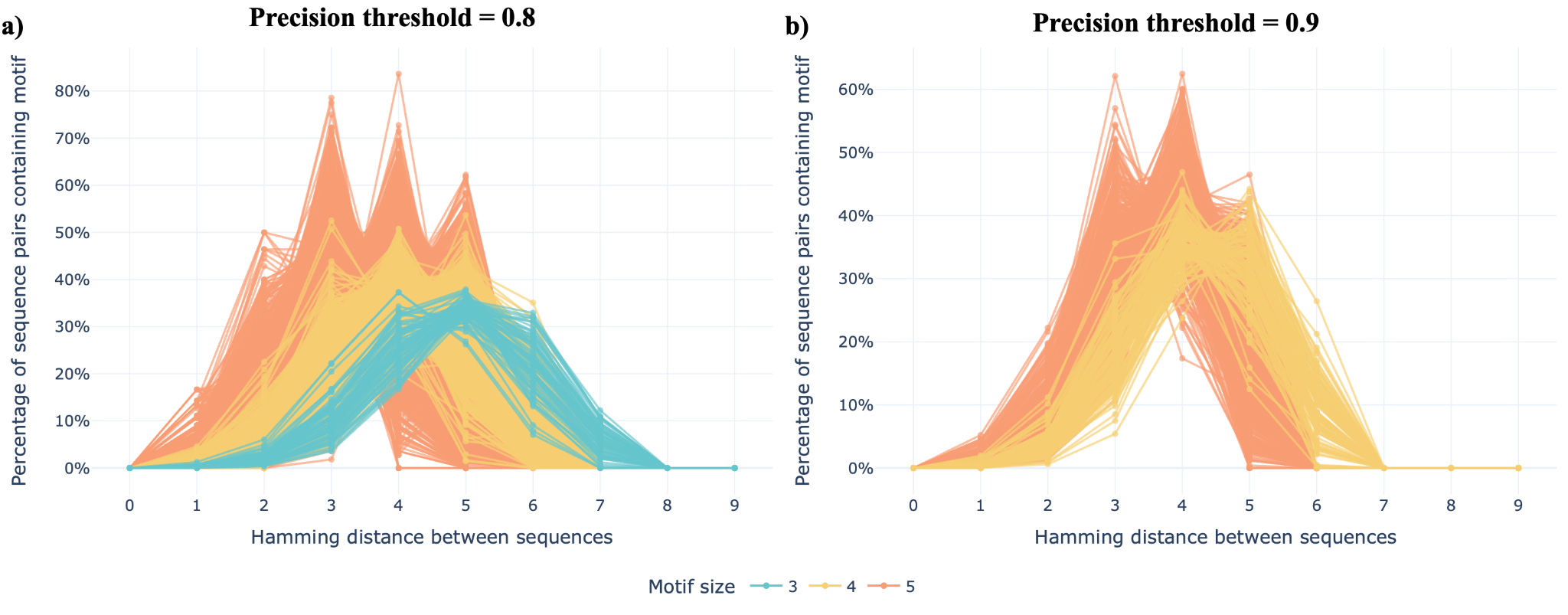
**

**Supplementary Figure 13: Sets of binder sequences containing the learned motifs show high overall dissimilarity.** The pairwise Hamming distance was computed between each pair of binder sequences in the Mason training + validation set containing each of the motifs learned using training precision thresholds 0.8 and 0.9. For the majority of sequence pairs containing a motif of interest, most of the non-motif positions contained different amino acids, meaning the motifs are able to capture groups of highly diverse binder sequences.


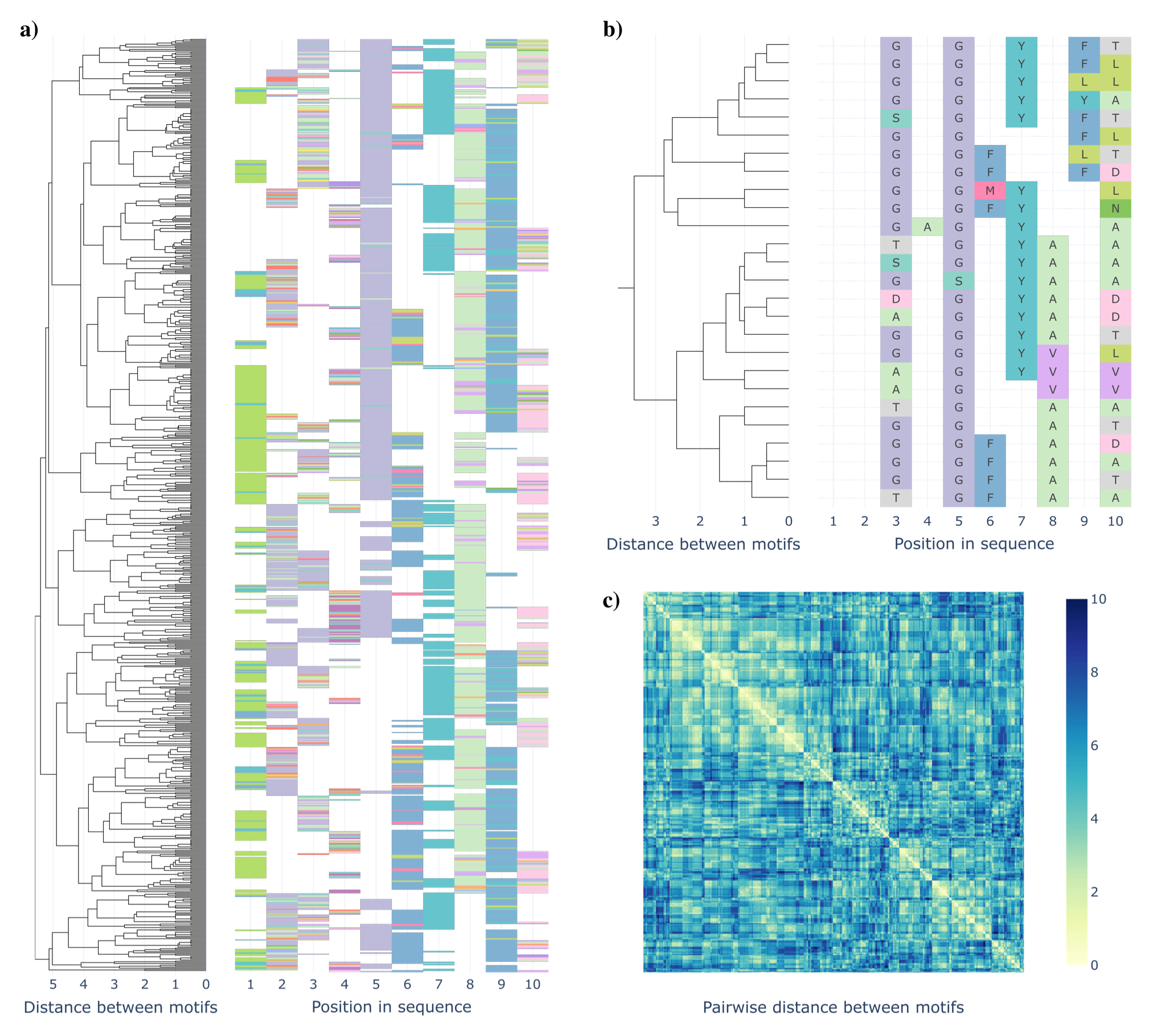


**Supplementary Figure 14: Clustering analysis of the motifs learned with training precision threshold 0.9.** Pairwise distances were computed between the set of 792 motifs, and UPGMA clustering was performed. The distance definition was as follows: a matching amino acid (or gap) between motifs counts as 0 distance, a gap in the position where the other motif has an amino acid is counted as 1 distance, and a mismatching amino acid in the same position counts as 0.5 distance. This groups motifs based on shared position usage, followed by amino acid usage. This figure shows (a) the full clustered set of 792 motifs, (b) an example of the first sub-cluster and (c) a heatmap of pairwise distances between all clustered motifs. The majority (564) of motifs had a neighbour within 0.5 distance, meaning only one amino acid was substituted, and the remaining motifs had a minimal distance of 1 (198 motifs) or 2 (24 motifs) to their nearest motif neighbour. While high-similarity sub-clusters can be found, the total set of motifs shows high overall diversity.

**Supplementary Table 9: The performance of each trained ML model on the Mason test set and independently generated Mehta test set.** The highest values in each column are bolded.

| Model | Accuracy | | Balanced accuracy | | Precision | | Recall | |
| --- | --- | --- | --- | --- | --- | --- | --- | --- |
|  | Mason | Mehta | Mason | Mehta | Mason | Mehta | Mason | Mehta |
| Mason CNN | **0.857** | 0.594 | **0.812** | 0.6 | 0.775 | 0.684 | 0.707 | 0.395 |
| Sequences with HD 1 | 0.74 | 0.494 | 0.551 | 0.508 | **0.859** | **0.793** | 0.109 | 0.023 |
| Sequences with HD 2 | 0.812 | 0.558 | 0.752 | 0.568 | 0.696 | 0.707 | 0.612 | 0.243 |
| Sequences with HD 3 | 0.633 | 0.599 | 0.724 | 0.597 | 0.435 | 0.602 | **0.935** | 0.65 |
| All motifs (precision > 0.8) | 0.724 | **0.677** | 0.777 | **0.675** | 0.51 | 0.665 | 0.9 | **0.751** |
| All motifs (precision > 0.9) | 0.813 | 0.598 | 0.726 | 0.606 | 0.75 | 0.763 | 0.522 | 0.317 |
| Selected motifs (precision > 0.8) | 0.824 | 0.633 | 0.781 | 0.639 | 0.698 | 0.742 | 0.681 | 0.441 |
| Selected motifs (precision > 0.9) | 0.808 | 0.581 | 0.704 | 0.591 | 0.78 | 0.777 | 0.46 | 0.261 |
| Motifs (precision > 0.8) + logistic regression | 0.836 | 0.584 | 0.767 | 0.593 | 0.772 | 0.738 | 0.605 | 0.298 |
| Motifs (precision > 0.9) + logistic regression | 0.807 | 0.574 | 0.696 | 0.584 | 0.794 | 0.775 | 0.439 | 0.242 |

**Supplementary Table 10: A confusion matrix comparing the predictions of the CNN and selected motif classifier (with training set precision threshold 0.8) on the Mason test set.** A similar table with results on the Mehta dataset is shown in Supplementary Table 11. While most of the predictions made by the models are the same (many TP and TN predictions in common), the mistakes made by both models were not notably similar. The number of times both models make the same wrong prediction (FP or FN) is not notably higher than the number of times one of the two models is wrong and the other correct.

|  | CNN TP | CNN TN | CNN FP | CNN FN | Row total |
| --- | --- | --- | --- | --- | --- |
| Selected motifs (precision > 0.8) TP | 1488 | 0 | 0 | 261 | 1749 |
| Selected motifs (precision > 0.8) TN | 0 | 5453 | 195 | 0 | 5648 |
| Selected motifs (precision > 0.8) FP | 0 | 424 | 332 | 0 | 756 |
| Selected motifs (precision > 0.8) FN | 329 | 0 | 0 | 491 | 820 |
| Column total | 1817 | 5877 | 527 | 752 | 8973 |

**Supplementary Table 11: A confusion matrix comparing the predictions of the CNN and selected motif classifier (with training set precision threshold 0.8) on the Mehta dataset.** A similar table with results on the Mason test set is shown in Supplementary Table 10. Note that the relatively higher fraction of (shared) FN predictions can be explained by the fact that the Mehta dataset is more balanced than the Mason test set, and thus contains fewer negative (non-binder) sequences.

|  | CNN TP | CNN TN | CNN FP | CNN FN | Row total |
| --- | --- | --- | --- | --- | --- |
| Selected motifs (precision > 0.8) TP | 53,500 | 0 | 0 | 22,346 | 75,846 |
| Selected motifs (precision > 0.8) TN | 0 | 121,835 | 13,922 | 0 | 135,757 |
| Selected motifs (precision > 0.8) FP | 0 | 8,863 | 17,566 | 0 | 26,429 |
| Selected motifs (precision > 0.8) FN | 14,521 | 0 | 0 | 81,782 | 96,303 |
| Column total | 68,021 | 130,698 | 31,488 | 104,128 | 334,335 |

**
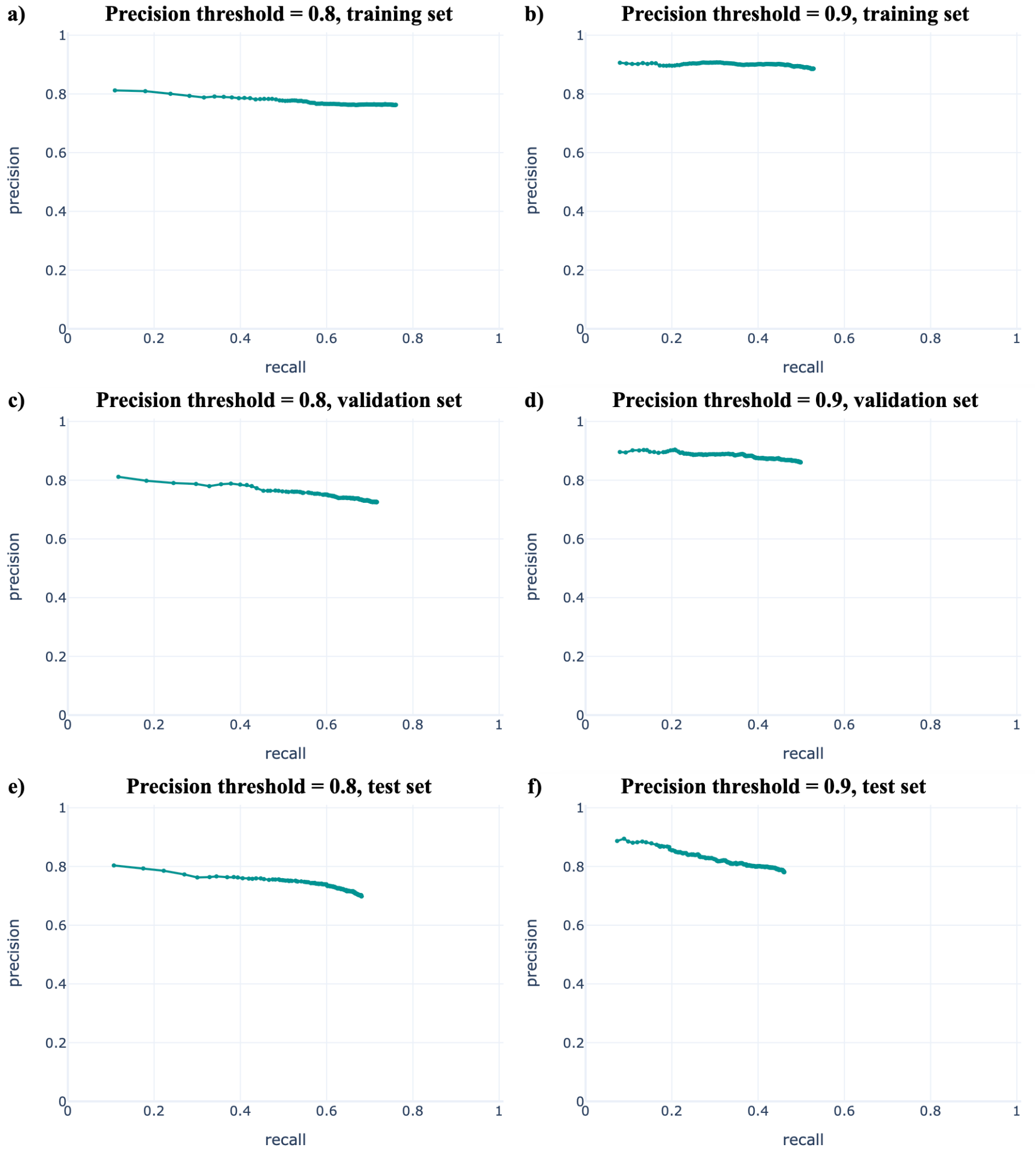
**

**Supplementary Figure 15: Precision and recall scores for each added motif to the collection of selected motifs.** The scores are shown for the (a, b) training, (c, d) validation and (e, f) test sets with training set precision thresholds 0.8 and 0.9, respectively. New motifs are greedily added to the collections based on which motif optimises the balanced accuracy on the training set, until the early stopping criterion (performance drop on the validation set) is reached. For training precision threshold 0.8, 178 out of the 11,336 motifs were selected, whereas for training precision threshold 0.9 this was 229 out of 792 motifs. While the number of learned motifs is lower with training precision threshold 0.8, the added recall from each of these motifs is higher.

**Supplementary Table 12: A comparison of gapped motifs to the most common k-mers as features for sequence classification.** To compare the power of high-precision (gapped) motifs versus (contiguous) k-mers selected purely based on frequency as ML features, two logistic regression models were trained. The first model using the set of *gapped* motifs learned with training precision threshold 0.9. Twelve motifs were removed from the original set of 791 motifs, due to being contiguous k-mers. The final set of features consisted of 106 and 674 gapped motifs of respective lengths 4 and 5. A second model was trained based on the most common position-specific contiguous k-mers across the Mason training + validation set. For fairness the number of features was matched, resulting in a combination of 106 4-mers and 674 5-mers. The results reported below show the performance on the Mason 25% test set and Mehta test set. On the Mason 25% test set, the gapped motifs show higher precision while the k-mers show higher recall. However, on the Mehta test set, gapped motifs outperform k-mers in both precision and recall, suggesting that k-mer based logistic regression may be more prone to overfitting to the distribution of the Mason dataset whereas the gapped motifs represent more generalisable patterns.

| Model | Precision | | Recall | |
| --- | --- | --- | --- | --- |
|  | Mason | Mehta | Mason | Mehta |
| Gapped motifs (precision > 0.9) + logistic regression | **0.793** | **0.776** | 0.438 | **0.242** |
| Most common k-mers + logistic regression | 0.727 | 0.655 | **0.541** | 0.229 |


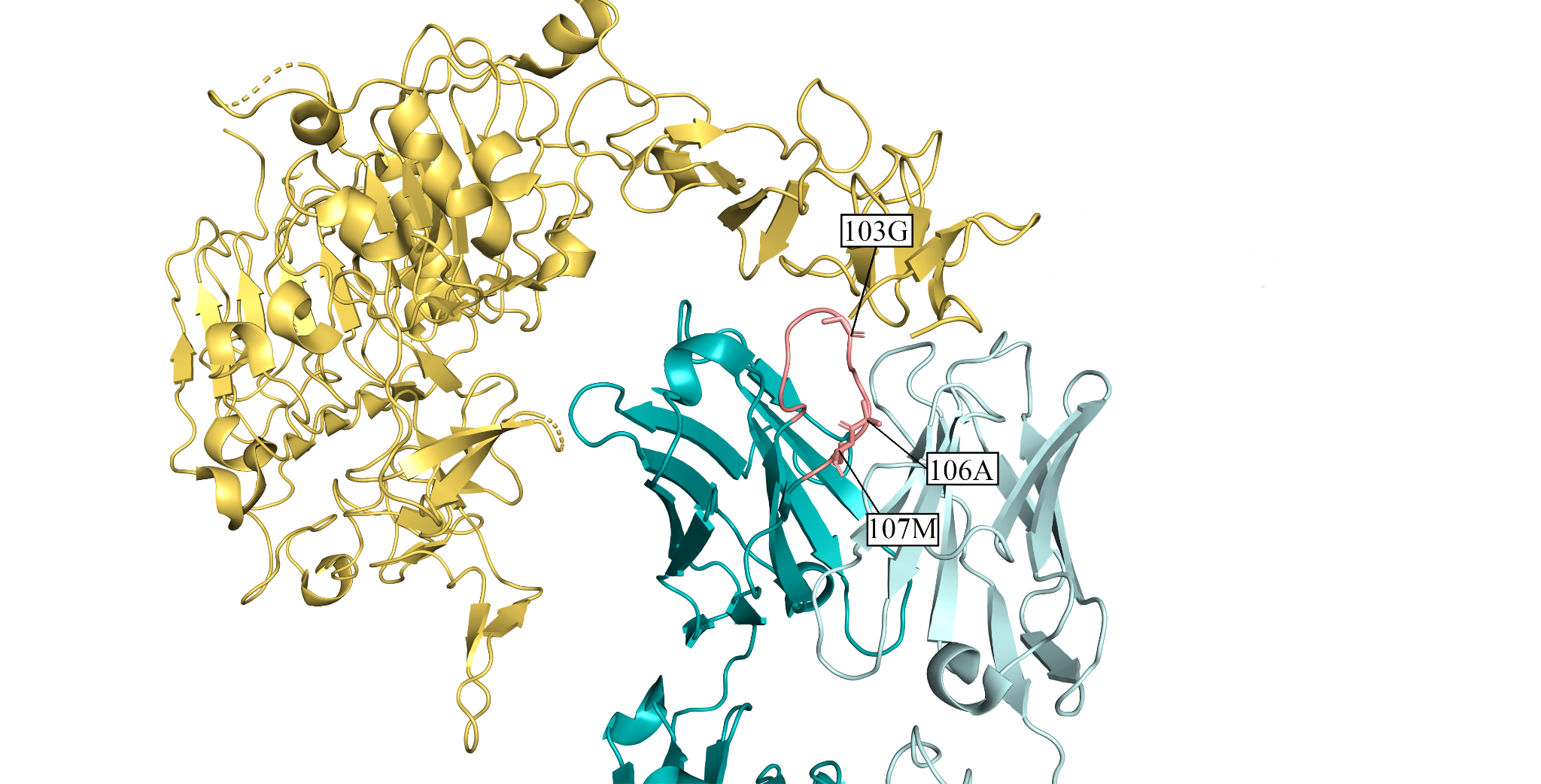


**Supplementary Figure 16: The 3D protein structure of Trastuzumab complexed with HER2.** Target antigen HER2 is shown in yellow, while the Trastuzumab antibody Fab fragment is shown in dark and light turquoise for the heavy and light chains respectively. The 10 amino acids in the CDRH3 which were varied across the dataset are highlighted in pink. The side chains of residues 103G, 106A and 107M are shown, which were the most frequently used positions in the antigen binding-associated motifs (positions 5, 8 and 9 in Supplementary Figure 11). While position 5 makes contact with the target antigen and is therefore part of the paratope, positions 8 and 9 are instead closer to the light chain in this structure. Of note, this structure shows only one 3D configuration of the antibody-antigen complex, and different variations of the trastuzumab antibody may differ in conformation and contact residues. This visualisation was made using PyMOL.
